# Autophagy-based unconventional secretion of HMGB1 in glioblastoma promotes chemosensitivity to temozolomide through macrophage M1-like polarization

**DOI:** 10.1186/s13046-022-02291-8

**Published:** 2022-02-22

**Authors:** Zhuang Li, Wen-Juan Fu, Xiao-Qing Chen, Shuai Wang, Ru-Song Deng, Xiao-Peng Tang, Kai-Di Yang, Qin Niu, Hong Zhou, Qing-Rui Li, Yong Lin, Mei Liang, Si-Si Li, Yi-Fang Ping, Xin-Dong Liu, Xiu-Wu Bian, Xiao-Hong Yao

**Affiliations:** 1 Institute of Pathology and Southwest Cancer Center, Ministry of Education of China, Southwest Hospital, Third Military Medical University (Army Medical University) and Key Laboratory of Tumor Immunopathology, Chongqing, China; 2 Department of Nephrology, Southwest Hospital, Third Military Medical University (Army Medical University), Chongqing, China; 3 Department of Oncology, Chinese Hainan Hospital of PLA General Hospital, Hainan Province, Sanya, China

**Keywords:** Glioblastoma, TMZ, Secretory autophagy, HMGB1, TAMs

## Abstract

**Background:**

Glioblastoma (GB) is the most common and highly malignant brain tumor characterized by aggressive growth and resistance to alkylating chemotherapy. Autophagy induction is one of the hallmark effects of anti-GB therapies with temozolomide (TMZ). However, the non-classical form of autophagy, autophagy-based unconventional secretion, also called secretory autophagy and its role in regulating the sensitivity of GB to TMZ remains unclear. There is an urgent need to illuminate the mechanism and to develop novel therapeutic targets for GB.

**Methods:**

Cancer genome databases and paired-GB patient samples with or without TMZ treatment were used to assess the relationship between HMGB1 mRNA levels and overall patient survival. The relationship between HMGB1 protein level and TMZ sensitivity was measured by immunohistochemistry, ELISA, Western blot and qRT-PCR. GB cells were engineered to express a chimeric autophagic flux reporter protein consisting of mCherry, GFP and LC3B. The role of secretory autophagy in tumor microenvironment (TME) was analyzed by intracranial implantation of GL261 cells. Coimmunoprecipitation (Co-IP) and Western blotting were performed to test the RAGE-NFκB-NLRP3 inflammasome pathway.

**Results:**

The exocytosis of HMGB1 induced by TMZ in GB is dependent on the secretory autophagy. HMGB1 contributed to M1-like polarization of tumor associated macrophages (TAMs) and enhanced the sensitivity of GB cells to TMZ. Mechanistically, RAGE acted as a receptor for HMGB1 in TAMs and through RAGE-NFκB-NLRP3 inflammasome pathway, HMGB1 enhanced M1-like polarization of TAMs. Clinically, the elevated level of HMGB1 in sera may serve as a beneficial therapeutic-predictor for GB patients under TMZ treatment.

**Conclusions:**

We demonstrated that enhanced secretory autophagy in GB facilitates M1-like polarization of TAMs to enhance TMZ sensitivity of GB cells. HMGB1 acts as a key regulator in the crosstalk between GB cells and tumor-suppressive M1-like TAMs in GB microenvironment and may be considered as an adjuvant for the chemotherapeutic agent TMZ.

**Supplementary Information:**

The online version contains supplementary material available at 10.1186/s13046-022-02291-8.

## Background

Glioblastoma (GB) is a heterogeneous and highly aggressive primary brain tumor with a 14.6-month patient survival [1, 2]. The standard treatment of GB consists of maximal resection followed by radiotherapy and concomitant chemotherapy with the alkylating agent temozolomide (TMZ) [3]. The addition of TMZ improved the overall survival and progression-free survival of GB patients compared with radiotherapy alone [1]. However, the effectiveness of TMZ is limited by drug resistance. Given the high mortality and relative resistance to conventional therapy, there has been significant interest in improving the understanding of the molecular landscape and treatment of GB.

Accumulating evidence indicates that autophagy as a lysosome-mediated process plays important roles during various stages of tumorigenesis [4]. Depending on the type of cancer and the surrounding tissue context, autophagy fulfils a dual task, having either tumor-promoting or suppressing properties. Autophagy is frequently activated as a stress response by tumor cells upon TMZ treatment [5]. Autophagy, which recycles breakdown products to sustain cell metabolism and biosynthesis under stress conditions, has been proposed as a mechanism of chemoresistance to alkylating drugs [6]. Combination of autophagy inhibitors, such as bafilomycin A1 and chloroquine (CQ), increases chemosensitivity to TMZ in glioma cell lines. However, the effect of autophagy inhibition in combination with TMZ did not improve the overall survival of patients with GB [5, 7]. The disputed effect of combination of autophagy inhibitors and TMZ in GB indicated that the role of autophagy in dependent on the context. Cytoprotective, cytotoxic, and cytostatic forms of autophagy induced by antitumor agents in various cancer models including GB have been reported [8–10]. The diverse roles of autophagy in cancer treatment have thus attracted considerable interest.

Recent research has revealed that certain leaderless proteins required autophagic membranes for efficient envelopment and exocytosis for unconventional secretion, named secretory autophagy [8, 9]. The role of autophagy in protein secretion and trafficking is recognized as a function of the autophagic machinery to expand the immediate sphere of influence from intracellular compartments to extracellular environments [10–12]. One of the major breakthroughs in this area is the recognition that a subset of unconventionally secreted cytosolic proteins, such as HMGB1, IL-18, and IL-1β, lack leader peptides and therefore cannot enter the endoplasmic reticulum-to-Golgi secretory pathway [13–15]. We have demonstrated that HMGB1 secreted by autophagic cancer-associated fibroblasts is critical for promoting the progression of luminal breast cancer [16]. HMGB1 is a highly conserved chromosomal protein and has multiple activities based on its location [17]. Inside the cells, HMGB1 sustains nucleosome dynamics and chromosomal stability and participates in DNA repair and telomere maintenance [18]. Outside the cells, HMGB1 interacts with multiple receptors to act as a cytokine and chemotactic cytokine to regulate inflammation and immunity [19, 20].

Extracellular HMGB1 has been reported to activate its receptors on GB cells through the downstream signaling pathway [e.g., NF-kB, IFN regulatory factor-3 (IRF3) and phosphoinositide 3-kinase (PI3K)] to produce a functional immune response, such as activation of tumor-associated dendritic cells (TADC), CD8^+^ T cells and macrophages [21, 22]. Besides, in the setting of bone marrow-derived macrophages, HMGB1 signaling by RAGE evoked NFκB activation in inflammatory reaction [17]. Abundant tumor associated macrophage (TAMs) infiltration is a common feature of GBs, but these TAMs lack apparent phagocytic activity. Recent studies demonstrated that TAMs in tumor microenvironment (TME) can be categorized into M1 and M2 subtypes based on their polarization status [23, 24]. In TME, the M1 or M2 subtype TAMs represent tumor-suppressive or tumor-supportive macrophages, respectively. M1-like macrophages exert cytotoxic activity on tumor cells and elicit tumor-destructive host reactions. M2-like TAMs are generally immune-suppressive and facilitate GB malignant behavior. TAMs are crucial players in tumor-host immune interaction and cancer progression. Cytokines released by tumor cells have been proved to regulate M1 or M2-like polarization [25, 26].

However, it is unclear whether any cytokines secreted by GB cells in TME through secretory autophagy induced by TMZ affect the tumor sensitivity to chemotherapeutic agent. In this study, we report a novel finding that autophagy-based unconventional secretion of HMGB1 in GB promotes chemosensitivity to TMZ through M1-like polarization of TAMs.

## Materials and methods

### Human GB specimens

Human GB specimens (*n* = 42) were obtained from Southwest Hospital, Third Military Medical University (Army Medical University) in Chongqing, China, with informed consent from patients or their guardians under an approved institutional review board protocol. Histopathological diagnosis was made by at least two neuropathologists based on the World Health Organization (WHO) classification. The clinicopathological characteristics of human GB specimens were summarized in Supplementary Table 1. Paired human GB samples (*n* = 41) are from patients with primary GB and its recurrent GB under TMZ treatment which were surgically excised at Southwest Hospital, Third Military Medical University, China. The tissues and sera of GB patients were saved at Southwest Hospital Biobank (No. [2021] BC0008) and regular follow-up was performed for patients. This work received approval from the ethics committee of Southwest Hospital, Army Medical University in Chongqing, China (KY2020294). Frozen GB sections and formalin-fixed, paraffin-embedded GB sections were stored at -20 °C or at room temperature, respectively. All procedures were performed in accordance with the principles of the Helsinki Declaration and approved by the institutional ethics committees.

### Bioinformatic analyses

All datasets were from the following public websites: The Cancer Genome Atlas (TCGA) (https://cancergenome.nih.gov/) and The Chinese Glioma Genome Atlas (CGGA) (http://www.cgga.org.cn/). Bioinformatic analysis plots were obtained from “GlioVis” (http://gliovis.bioinfo.cnio.es/) [27], a website for data visualization and analysis to explore gene expression data from studies involving patients with brain tumors.

GB scRNA-seq dataset was downloaded from GEO datasets (GSE84465). Raw matrix was preprocessed using computational methods deposited in the Seurat R package. Quality control was performed to ensure that only high-quality single-cell data was processed further, and cells with fewer than 4000 genes/cell and fewer than 8000 UMIs/cell were eliminated. Cells with greater than 20% of their transcriptome represented in mitochondrial transcripts were also excluded. We used Seurat v3 method in R v3.6 for data normalization, dimensionality reduction and clustering by default parameters. Cell types were annotated using canonical lineage makers and the expression of genes for *TLR2, TLR4, TLR9, RAGE* were visualized using violin polts.

### Cell culture

Primary human GB1 (090116) and GB2 (20171016B) cells as well as GB cell lines U251, LN229 and mouse GB cell line GL261 were cultured in Dulbecco’s Modified Eagle’s Medium (DMEM) (Gibco). The primary human GB cells (GB1 and GB2) were generated in our laboratory [28–30], which isolated from two surgical specimens of GB patients (Southwest Hospital, AMU, China). The specimens were cut into 1 × 1 × 1 mm^3^ pieces and cultured in 0.2 ml FBS in a 60 mm dish inverted for 1 h. The dish was then turned the right side up and supplemented with 5 ml DMEM containing 10% FBS. The primary GB cells were detached with 0.25% trypsin upon formation of a monolayer of cells.

Human THP-1-dreived macrophage and mouse Raw264.7 macrophage were cultured in RPMI 1640 medium (RPMI) (Gibco) and human microglia HMC3 was in MEM medium (Gibco) containing 10% FBS (Gibco) and 1:100 penicillin–streptomycin (Hyclone) at 37 °C in 5% CO_2_.

### Reagents

TMZ (No. PHR1437, Sigma) was dissolved in dimethyl sulfoxide (DMSO) at a 100 mM stock concentration and stored at -20 °C. Autophagy inhibitor 3-MA (No. M9281, Sigma) and LY294002 (No. S1105, Selleck Chemicals) were dissolved to 10 mM and 100 μM as stock solutions. CY-09 (No. S5774, Selleck Chemicals) and FPS-ZM1 (No. S8185, Selleck Chemicals) were at 5 mM and 100 μM as stock concentrations. Recombinant human HMGB1 (rhHMGB1, No.1690-HMB-050) was purchased from R&D Systems and recombinant mouse HMGB1 (rmHMGB1, Cat. 50913-M01H) was from Sino Biological.

### qRT-PCR

qRT-PCR was performed as previously described [16]. The specific primers for amplification were listed in Supplementary Table 2. All experiments were performed in quadruplicate samples.

### Co-immunoprecipitation (CO-IP) and immunoblotting

For CO-IP, cells were lysed in Pierce IP lysis buffer (No. 87787, Thermo Fisher) and protease inhibitor cocktail (No. 04693159001, Roche). Cell lysates (500 μl) were incubated with anti-HMGB1 antibody (ab18256, 1:200, Abcam) or control IgG (40 μl Protein A/G PLUS-Agarose, No. 17061801, GE health) overnight at 4 °C. The beads were washed three times with PBS, followed by immunoblotting. Immunoblotting was performed as previously described [31]. Primary antibodies used were anti-HMGB1 (ab18256, 1:1000, Abcam), anti-SQSTM1/p62 (88588S, 1:1000, CST), anti-ATG5 (12994 T, 1:1000, CST), anti-ATG7 (8558 T, 1:1000, CST), anti-LC3B (sc-376404, 1:1000, Santa), anti-GAPDH (5174S, 1:1000, CST), anti-RAGE (ab3611, 1:1000, Abcam), anti-GORASP2 (10598–1-AP, 1:1000, Proteintech), anti-p-ERK1/2 (8544S, 1:1000, CST), anti-IKB (9246S, 1:1000, CST), anti-NLRP3 (19771–1-AP, 1:1000, Proteintech), anti-ASC (sc-514414, 1:1000, Santa) and anti-p-NF-κB p65 (3033S, 1:1000, CST), anti-β-TUBULIN (2128S,1:1000, CST).

### Immunohistochemistry (IHC)

Tissue slices were deparaffinized and hydrated by a series of xylene and alcohol treatment. The slices were incubated with anti-HMGB1 (ab18256, 1:200, Abcam), anti-LC3B (3868S, 1:200, CST), anti-SQSTM1/p62 (88588S, 1:200, CST) and anti-Syntaxin 17 (STX17, 17815–1-AP, 1:100, Proteintech) at 4 °C overnight, followed by incubation with avidin–biotin-peroxidase. HMGB1 was considered positive by nucleus staining and the expression levels were semi-quantified by a composite score system based on both the percentage and intensity of stained tumor cells. The percentage of positive cells was calculated in high-power fields (HPF) as follows: 0 (< 10% positive tumor cells), 1 (11%-50% positive tumor cells), 2 (51%-75% positive tumor cells) and 3 (> 75% positive tumor cells). The staining was scored by two independent neuropathologists as the proportion of positive tumor cells × the staining intensity. Images were captured using a DP72 digital camera (Olympus) connected with a BX51 microscope (Olympus).

For calculating integrated optic density (IOD) values of LC3B, SQSTM1, STX17 and HMGB1 in tumor cells, five representative fields (magnification × 200) in the region of tumor cells were randomly selected. Image‐Pro Plus 6.0 software (MEDIA Cybernetics, Rockville, MD, USA) was utilized to measure the areas of tumor cell region excluding stroma areas and the IOD of the tumor cells expressing LC3B, SQSTM1, STX17 and HMGB1. Recognition of tumor cell regions was conducted under the guidance and confirmation of pathologists.

### Immunofluorescence (IF)

Mouse GB xenografts were collected from mice when neurological signs occurred after GB cell transplantation. Human GB specimens were obtained from patients through surgical resection. Cultured cells or tumor sections were fixed in 4% paraformaldehyde for 15 min and washed with PBS twice. Samples were blocked with PBS containing 1% BSA plus 0.3% Triton X-100 for 30 min at room temperature, then incubated with indicated primary antibodies overnight at 4 °C followed by the fluorescent second antibody at room temperature for 1 h. Nuclei were counterstained with DAPI for 5 min, and then sections were mounted on glass and subjected to microscopy. Primary antibodies listed as follows: anti-CD206 (sc-58986, 1:200, Santa), anti-CD206 (MCA2235, 1:200, Bio-Rad), anti-IBA1 (ab5076, 1: 100, Abcam), anti-IBA1 (019–19741, 1:400, Wako), anti-CD16/32 (553142, 1: 200, BD Biosciences), anti-LC3B (3868S, 1: 200, CST), anti-TLR2 (JM22-41, 1:200, Thermo Fisher), anti-TLR4 (ab22048, 1: 100, Abcam), anti-TLR9 (IMG-305A, 1: 100, NOVUS), anti-RAGE (sc-365154, 1:100, Santa), anti-HMGB1 (ab18256, 1:100, Abcam), anti-HMGB1 (sc-56698, 1:100, Santa), anti-TNF-α (MAB610, 1: 200, NOVUS), anti-IFN-γ (8455S, 1: 200, CST), anti-IL-1β (12703S, 1: 200, CST), anti-IL-6 (ab9324, 1: 100, Abcam), anti-IL-8 (MAB208, 1: 200, NOVUS), anti-CCL2 (39091S, 1: 200, CST), anti-NLRP3 (19771–1-AP, 1:100, Proteintech) and anti-ASC (sc-514414, 1:200, Santa).

### Transmission electron microscopy (TEM)

GB cells were harvested after drug treatments, washed twice with PBS, and fixed with ice-cold-glutaraldehyde (3% in 0.1 M cacodylate buffer, pH 7.4) overnight. Thin Sects. (75 nm) were prepared, stained with methylene and observed under light microscopy. All ultra-thin sections were stained with lead citrate for observation under TEM with a Hitachi HT7700 electron microscopy (Tokyo, Japan) at 80 kV.

### Assay for autophagy

GB cells in 15 mm confocal dish plates were infected with AAV-mCherryGFP-LC3B (MOI = 30, HB-AP2100001, Hanbio) according to the manufacturer’s instruction. After 24 h, cells were treated with agents as required. Six fluorescence fields were captured with confocal microscopy (LSM900, ZEISS). Green and red fluorescence represented phagophore and autolysosome, respectively and yellow puncta represented autophagosome. The autophagic activity indicated by formation of autophagosome was determined by quantifying the yellow puncta. Six fields per sample and three replicates per treatment were included.

### Gene silencing with siRNA and lentiviral transduction

GB cells were seeded at 5** × **10^4^ cells/well in 6-well plates. For gene knockdown, GB cells were transfected with Lipofectamine RNAiMAX (No. 13778150, Invitrogen). siRNA and Lipofectamine RNAiMAX reagent were diluted into Opti-MEM medium and mixed at a 1:1 ratio, which were incubated for 5 min at room temperature before adding to the cells. For *ATG*5 and *GORASP*2 depletion, cells were analyzed for 48 h after specific siRNA transfection. siRNA for human *ATG*5 (Signal Silence® Atg5 siRNA I #6345) and control (Signal Silence® Control siRNA #6568) was purchased from Cell Signaling Technology. siRNA for human *GORASP*2 was purchased from RiboBio Technology. Human *HMGB1*/mouse *Hmgb1*-specific shRNA and shCtrl vectors were purchased from Hanbio Technology Corp. Ltd., China. Lentivirus packaging and transduction were conducted as described previously [31]. shRNA sequences are listed in Supplementary Table 3.

### ELISA

Concentrations of HMGB1 in the culture supernatant of GB cells were determined using a Human ELISA Kit (#ARG81185, Arigo) according to the manufacturer’s instructions. IL-33, IL-1α, IL-37, IL-18, FGF1, FGF2, Galectin-3, MIF, Annexin A1, S100A8, IL-6 and CCL2 were measured by ELISA Kit (#EK0929, #EK0389, #EK1363, #EK0864, #EK0339, #EK0342, #EK0764, #EK0813, #EK1745, #EK1558, #EK0410, #EK0441, BOSTER). HMGB1 in GB patient sera was measured using an ELISA Kit (#6010, Chondrex). TNF-α, IL-8 and IFN-γ (#EL10019, #EL10008 and #EL10024, Anogen) and IL-1β (#DLB50, R&D) were measured by ELISA.

### Co-culture

THP1 cell line-derived macrophages (1** × **10^5^) and GB cell line LN229 (1** × **10^5^) were cultured in the upper and lower chamber of Transwell Permeable Support systems (No. 353493 Corning) with a 0.4 µm pore-size filter. All co-culture was conducted in the presence of TMZ (1000 μM) for 24 h.

### Cell viability

For CCK-8 assay, cells were inoculated in 96-well plates and maintained in an incubator for time period. CCK-8 solution (10 μL, B34304, Bimake) was added to each well for 1.5 h prior to measurement at OD 450 nm.

### Orthotopic xenograft

GL261 cells (5** × **10^4^) with pLVX-puro-linker-luciferase lentivirus were injected intracranially into the right frontal lobes of 4–6-week-old male C57BL/6 mice (Laboratory Animal Center, Southwest Hospital, Third Military Medical University (Army Medical University), China). Mice were grouped by 10 animals in large plastic cages and were maintained under pathogen-free conditions. Growing xenograft tumors were detected and quantified by bioluminescence imaging using an In Vivo Image System (IVIS) (PerkinElmer, USA). Mice with neurological signs or moribund were sacrificed and tumors were collected. The animal experiments were approved by the Institutional Animal Care and Use Committee of Southwest Hospital, Third Military Medical University (Army Medical University) in accordance with the Guide for the Care and Use of Laboratory Animals.

### Tissue dissociation and flow cytometry analysis

GL261 cell-derived xenograft mice with different treatment were perfused transcardially with cold phosphate-buffered saline (PBS) to clear away blood cells from the brain. The tumor-bearing brain hemispheres were dissociated enzymatically to obtain a single-cell suspension with a Brain Tumor Dissociation Kit (No. 130–095-942, Miltenyi Biotec) according to the manufacturer’s protocol. The cell suspension was filtered through a 70 μm strainer and centrifuged at 300 × g, 4 °C for 10 min. Next, myelin was removed by centrifugation on 30% Percoll gradient (No. 17089101, GE Healthcare). Cells suspension was centrifuged at 1050 × g, 4 °C for 30 min without acceleration and brakes. Finally, cells were collected for flow cytometric analysis. Cells were incubated for 15 min with True-Stain Monocyte Blocker™ (No. 426102, Biolegend) in FACS Buffer to block FcγRIII/II and reduce unspecific antibody binding. For surface marker analysis, cells were re-suspended in FACS buffer and stained with PerCP-Cyanine5.5 anti-CD45 (103132, 1:100, Biolegend), APC anti-CD11b (101212, 1:100, Biolegend), PE/Cyanine7 anti-CD86 (105013, 1:100, Biolegend), and PE anti-CD11c (117308, 1:100, Biolegend) at 4 °C for 30 min. Cells were fixed and permeabilized (No. 554714, BD) for intracellular protein staining, then labeled with FITC anti-CD206 (141704, 1:100, Biolegend). Data were acquired by the BD LSRFortessa and analyzed with FlowJo software v10.

### Statistical analysis

Similar results were obtained from three independent experiments. Statistical differences were determined by two tailed unpaired Student’s t-test for two groups or by two-way ANOVA for multiple groups. Results presented in this study are as the mean ± SD. For Kaplan–Meier survival curves and statistical differences were determined by log-rank test. All analyses were carried out using Microsoft excel 2019 or GraphPad Prism 8.3 software. *P* < 0.05 was considered statistically significant. Detailed information is described in Figure legends. Significant statistical differences were defined as *, *P* < 0.05; **, *P* < 0.01; ***, *P* < 0.001.

## Results

### Exocytosis of HMGB1 induced by TMZ is dependent on the formation of autophagic vacuoles

To determine whether TMZ promoted the exocytosis of leaderless proteins, the secretory autophagy proteins HMGB1, IL-33, Galectin-3, IL-1α, MIF, FGF1, AnnxinA1, S100A8, IL-1β, IL-37, IL-18 and FGF2, in supernatants of primary GB cells (GB1 and GB2 cells) and cell lines (U251 and LN229) under TMZ were analyzed. HMGB1 is significantly elevated in supernatants with TMZ than in control without TMZ (Fig. S1). The mRNA of autophagy-related genes *ATG5* (autophagy-related 5), *ATG7* (autophagy-related 7)*, LC3B* (microtubule associated protein 1 light chain 3 beta) and *BECN1* were elevated in GB cells under TMZ treatment (Fig. S2A).

Primary GB1 and U251 cells were engineered to express an autophagic flux reporter protein consisting of mCherry, GFP and LC3B. As expected, TMZ enhanced the autophagic activity indicated by yellow autophagosome puncta (Fig. S2B-C) and the formation of autophagic vacuoles (Fig. S2D) in these cells.

**Fig. 1:**
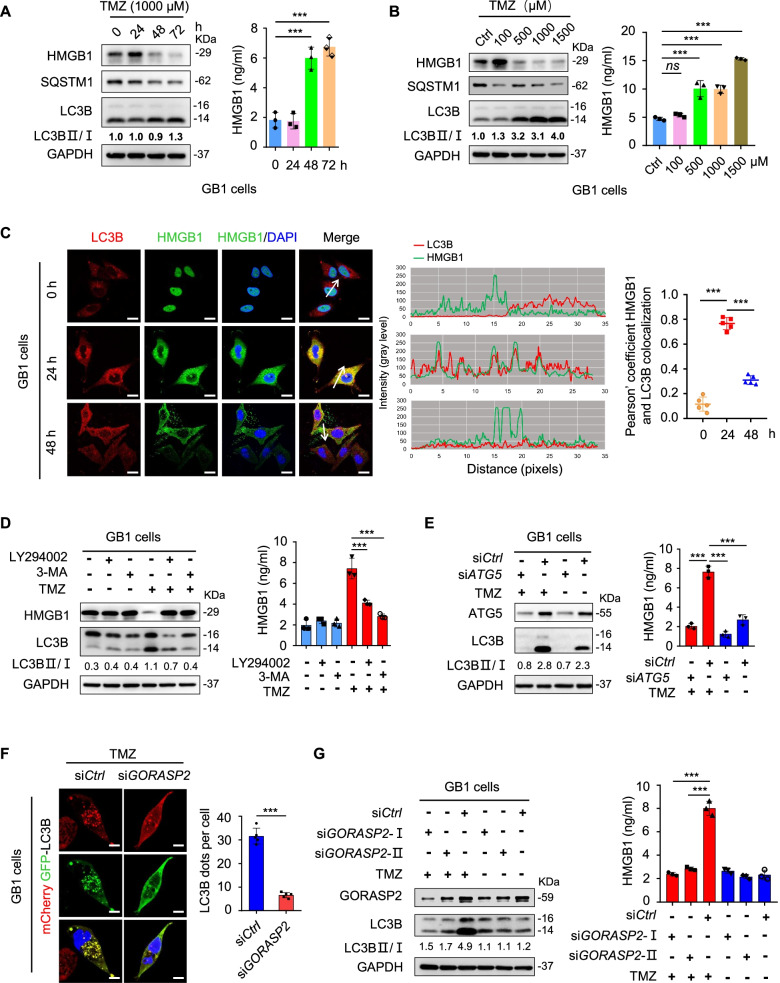
Release of HMGB1 induced by TMZ in GB is dependent on the autophagic vacuoles. **A** Immunoblot of HMGB1, SQSTM1 and LC3B-I to LC3B-II conversion in primary GB1 cells (GB1 cells) treated with 1000 μM TMZ (left panel). ELISA determining the HMGB1 in supernatants of primary GB1 cells (GB1 cells) (right panel). *n* = 3. **B** Immunoblot of HMGB1, SQSTM1 and LC3B-I to LC3B-II conversion in GB1 cells treated with TMZ for 72 h (left panel). ELISA determining the HMGB1 in supernatants of GB1 cells (right panel). *n* = 3. **C** Colocalization of LC3B (red) with HMGB1 (green) in GB1 cells under TMZ (left panel). Colocalization tracer profile along the line (white arrows) is indicated as merged images (middle panel). Pearson’s colocalization coefficient for LC3B and HMGB1 derived from three independent experiments with five fields (right panel). Scale bars = 10 μm. **D** Immunoblot of HMGB1 and LC3B-I to LC3B-II conversion in GB1 cells with or without 3-MA (5 mM) and LY294002 (100 nM) for 4 h, then stimulated with or without TMZ (1000 μM) for 24 h (left panel). HMGB1 was detected in the supernatants by ELISA (right panel). **E** Immunoblot of ATG5 and LC3B-I to LC3B-II conversion in GB1 cells transiently transfected with scrambled siRNA (si*Ctrl*) or siRNA *ATG5* (si*ATG5*) for 48 h then treated with TMZ (1000 μM) for 24 h (left panel). HMGB1 in the supernatants was detected by ELISA (right panel). **F** GB1 cells transfected with scrambled siRNA (si*Ctrl*) or siRNA *GORASP2* (si*GORASP2*) for 36 h were transfected with AAV-mCherryGFP-LC3B then treated with TMZ (1000 μM) for 24 h (left panel). The number of autophagosomes was analyzed in ten random fields for each independent experiment (right panel). **G** Immunoblot of GORASP2 and LC3B-I to LC3B-II conversion in GB1 cells transiently transfected with si*Ctrl* and si*GORASP2* for 48 h then with TMZ (1000 μM) treatment for 24 h (left panel). HMGB1 in the supernatants was detected by ELISA (right panel). *P < 0.05, **P < 0.01,***P < 0.001, *ns* = no significance

To further detect the autophagy activity of GB cells under TMZ, we used Western blot to observe the level of LC3B and SQSTM1. LC3B, which is required for the elongation of autophagosomes and widely used as a biomarker of autophagy, has two forms: type I is cytosolic and type II is cleaved, lipidated and membrane-bound. During autophagy activation, LC3B-II increases due to the conversion of LC3B-I. The ratio of LC3B-II to LC3B-I is considered the most reliable marker for quantification of the level of autophagy in cells. SQSTM1, another autophagy-related protein, is incorporated into the completed autophagosome and is degraded in autolysosomes, thus serving as an index of autophagic degradation. The decreased SQSTM1 levels are associated with autophagy activation [32]. When autophagy activity was elevated (the increase of LC3B-I to LC3B-II conversion and the decrease of SQSTM1) by TMZ treatment, the intracellular HMGB1 in GB cells decreased and the extracellular HMGB1 increased (Fig. 1A-B and Fig. S2E). HMGB1 was also visualized to translocate time-dependently from the nuclei to the cytoplasm and extracellular space with TMZ treatment as measured by immunofluorescence confocal microscopy (Fig. 1C, left panel). Pearson’ colocalization analysis showed that HMGB1 and LC3B were colocalized (Fig. 1C, middle and right panel). Addition of autophagy inhibitors, 3-methyladenine (3-MA) and LY294002 reduced LC3B-I to LC3B-II conversion induced by TMZ associated with significant decrease in extracellular HMGB1, which was retained in intracellular regions (Fig. 1D and Fig S3A).

We additionally knocked down ATG5, a core molecular machinery component involved in autophagosome formation [33], to test the effect on secretory autophagy by GB cells (Fig. S3B). Knockdown of ATG5 in GB cells (si*ATG5*) resulted in a marked decrease in LC3B-I to LC3B-II conversion and significant reduction of extracellular HMGB1 upon TMZ treatment (Fig. 1E and Fig. S3C). Previous studies have demonstrated that the golgi reassembly stacking protein 2 (GORASP2) facilitates autophagosome-lysosome fusion and controls the secretory autophagy [34, 35]. We thus transiently transduced GB cells with adenovirus harboring si*RNA* against GORASP2 (si*GORASP2-*I and II) and a scrambled si*RNA* sequence (si*Ctrl*) as a control. The autophagic flux (autophagosomes and autolysosomes) decreased in si*GORASP2-*I and II GB cells transfected with AAV-mCherryGFP-LC3B upon TMZ treatment (Fig. 1F). Western blot showed that knockdown of *GORASP*2 in GB cells reduced LC3B-I to LC3B-II conversion and extracellular levels of HMGB1 promoted by TMZ (Fig. 1G and Fig. S3D). These data suggest that the exocytosis of HMGB1 induced by TMZ in GB cells is dependent on the formation of autophagic vacuoles.

### Extracellular HMGB1 serves as a TMZ therapeutic predictor for GB patients

**Fig. 2:**
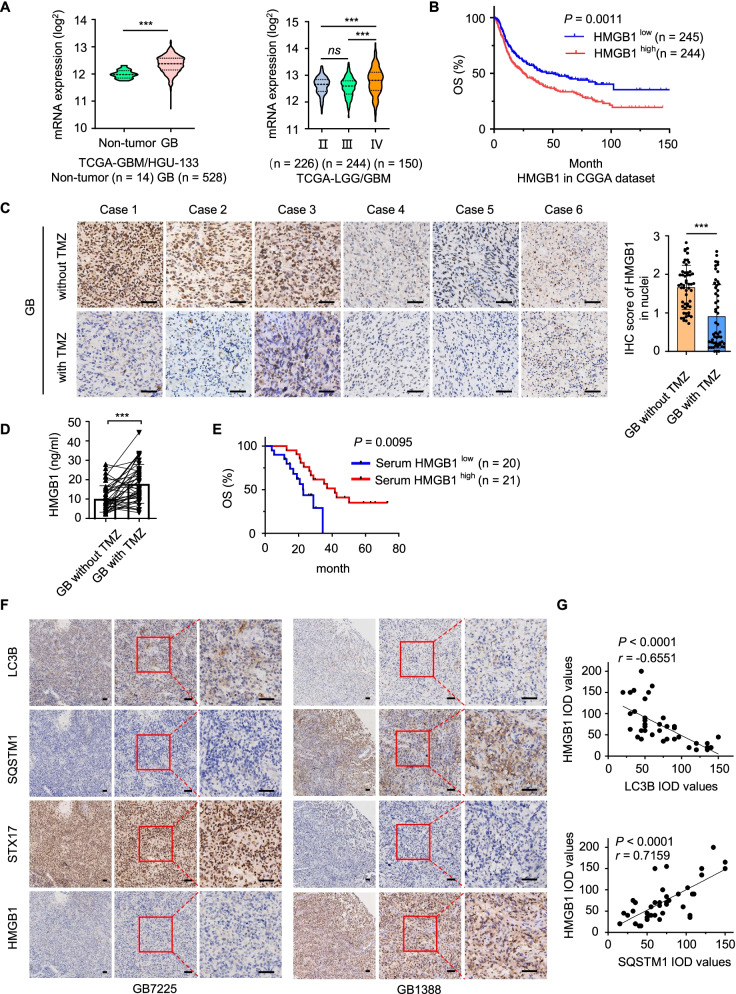
The correlation between HMGB1 levels and the prognosis of GB patients treated with TMZ. **A** The mRNA levels of HMGB1 in non-tumor tissues (*n* = 14) and GB tissues (*n* = 528) from TCGA-GBM/HGU133 datasets (left panel), as well as in WHO II (*n* = 226), WHO III (*n* = 244) and WHO IV (*n* = 150) grade glioma tissues from TCGA-LGG/GBM datasets (right panel). **B** The overall survival (OS) rates of mRNA HMGB1^low^ and HMGB1^high^ GB patients from CGGA datasets (*n* = 489). **C** Representative immunohistochemistry (IHC) of HMGB1 in paired GB patients (left panel) with quantification (right panel). HMGB1 stains brown color. *n* = 41, Scale bars = 50 μm. **D** The concentration of HMGB1 in sera of paired GB patients. *n* = 41. **E** The OS rate of TMZ-treated serum HMGB1^low^ and HMGB1^high^ GB patients. *n* = 41. **F** Representative IHC of LC3B, SQSTM1, STX17 and HMGB1 in serial sections of GB specimens (GB7225 and GB1338) with TMZ treatment. Areas examined under higher magnification were marked. Brown color is positive. Scale bars = 50 μm. **G** The correlation between LC3B and HMGB1 expression in GB patients with TMZ treatment (*n* = 41) (upper panel). The correlation between SQSTM1 and HMGB1 expression in GB patients with TMZ treatment (*n* = 41) (lower panel). Pearson’s correlation test. *P < 0.05, **P < 0.01,***P < 0.001, *ns* = no significance

To investigate the function of HMGB1 in GB patients, we analyzed mRNA levels of HMGB1 in GB and non-tumor tissues from the TCGA-GBM and Rembrandt dataset. Compared to non-tumor and low-grade glioma, mRNA levels of HMGB1 were significantly increased in GB (Fig. 2A and Fig. S4A-B). GB with high HMGB1 expression in intracellular region (including the nucleus and cytoplasm) predicted a worse overall survival rate (OS) of patient than those with lower HMGB1 expression in the tumors (Fig. 2B). The results are consistent with the contribution of intracellular HMGB1 to DNA repair and tumorigenesis [36, 37].

To study the role of extracellular HMGB1 in GB, we collected tissues and sera of 41 patients with paired GBs. IHC staining showed that HMGB1 was mainly located in the nuclei of GB tissues without TMZ treatment, and in paired GBs with TMZ treatment, HMGB1 was positioned in the extracellular region (Fig. 2C, left panel). Quantitative analysis showed that the IHC scores of intracellular HMGB1 were significantly reduced in GB tissues upon TMZ treatment (*n* = 41) (Fig. 2C, right panel). We further measured the HMGB1 in sera of paired GB patients. Compared with GBs without TMZ, the levels of HMGB1 in sera significantly enhanced in GBs with TMZ treatment (Fig. 2D). Therefore, TMZ cause the translocation of HMGB1 from intracellular to extracellular regions in GB. Kaplan–Meier analysis demonstrated that GB patients who received TMZ treatment with high HMGB1 in sera displayed a favorable OS than those with lower HMGB1 in sera (Fig. 2E). Therefore, our studies indicate that HMGB1 in the intracellular region of GB is detrimental to the survival of patients. However, when HMGB1 is released into the extracellular space upon TMZ, it might be beneficial for GB patients. Thus, HMGB1 based on its location may function as a prognostic and treatment-predictor for GB patients.

To determine the correlation between autophagy and HMGB1 in GB tissues, we observed the expression of autophagy proteins and the location of HMGB1 in human GB specimens (GB7225, GB1388) with TMZ treatment. In GB7225 specimen with high level of autophagy (higher expression of LC3B, STX17 and lower expression of SQSTM1), HMGB1 in the nuclei and cytoplasm of tumor cells was significantly diminished. However, in GB1388 specimen with lower levels of autophagy (lower expression of LC3B, STX17 and higher expression of SQSTM1), HMGB1 accumulated in the nuclei and cytoplasm of tumor cells (Fig. 2F-G). These results suggest that extracellular HMGB1-dependent secretory autophagy prolonged the overall survival of GB patients, which may elevate the sensitivity of TMZ. This is consistent with recent reports showing that extracellular HMGB1 acting as a cytokine or chemotactic cytokine stimulates antitumor immunity responses during chemotherapy or radiotherapy [38–40].

### HMGB1 contributes to M1-like polarization of macrophages in GB

**Fig. 3:**
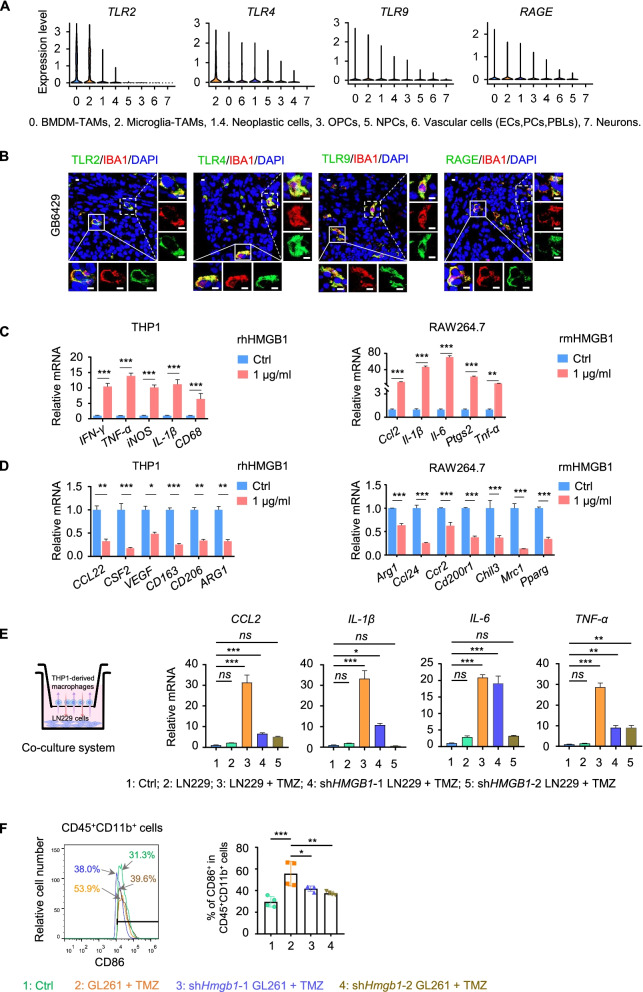
HMGB1 contributes to M1-like polarization of macrophages. **A** The mRNA expression of HMGB1 receptors (*TLR2, TLR4, TLR9* and *RAGE*) in human GB specimens from GEO database (Bone marrow derived macrophage-tumor associated macrophages, BMDM-TAMs; Macroglia-TAMs; Neoplastic cells; Oligodendrocyte progenitor cells, OPCs; Neural progenitor cells, NPCs; Endothelial cells, ECs; Pericytes, PCs; Peripheral blood lymphocytes, PBLs; Neurons.) was analyzed by single cell sequencing. **B** Immunofluorescence (IF) of HMGB1 receptors TLR2, TLR4, TLR9 and RAGE (green) in TAMs marked by IBA1 (red) in human GB samples (GB6429) without TMZ. Scale bars = 10 μm. **C** The mRNA level of M1-like phenotype genes in THP1 cell line-derived and RAW264.7 macrophages stimulated with 1 μg/ml recombinant human HMGB1 (rhHMGB1) or recombinant mouse HMGB1 (rmHMGB1) for 24 h. **D** The mRNA level of M2-like phenotype genes in THP1 cell line-derived and RAW264.7 macrophages stimulated with 1 μg/ml rhHMGB1 or rmHMGB1 for 24 h. **E** A scheme for in vitro co-culture system (left panel). THP1 cell line-derived macrophages (upper chamber) and sh*HMGB*1 or sh*Ctrl*-LN229 cells (low chamber) were co-cultured for 24 h then with TMZ treatment for 24 h. The expression of M1-like phenotype genes, *CCL2, IL-1β, IL-6* and *TNF-α*, was detected by qPCR (right panel). **F** Representative flow cytometric analysis of CD86^+^ TAMs gated on live CD45^+^ CD11b^+^ TAMs in GL261 cell-derived xenograft tumors with treatment of Ctrl (C), TMZ (T), rmHMGB1 (H) and TMZ plus rmHMGB1 (T + H) for 21 days (left panel). The histogram showed statistical analysis (right panel). *n* = 4. *P < 0.05, **P < 0.01, ***P < 0.001, *ns* = no significance

HMGB1 interacts with receptors to act as a cytokine or chemokine to regulate inflammation and immunity. Reported receptors for HMGB1 included TLR2, TLR4, TLR9 and RAGE [17, 38–40]. We analyzed the expression of these putative HMGB1 receptors in human GB specimens by single cell sequencing and found that these molecules mainly located in tumor associated macrophages (TAMs) in the microenvironment (Fig. 3A).

Previous studies have demonstrated that tumor microenvironment (TME) of GBs contains abundant TAMs, including both tumor-supportive macrophages (M2-like TAMs) and tumor-suppressive macrophages (M1-like TAMs) [41]. Immunofluorescence showed that TAMs marked by IBA1 in TME of human GB samples (GB6429) without TMZ treatment expressed HMGB1 receptors TLR2, TLR4, TLR9 and RAGE (Fig. 3B). We then cultured human THP1 cell line-derived macrophages, mouse RAW264.7 macrophages and human microglia HMC3 with rhHMGB1 or rmHMGB1. The mRNA levels of macrophage subpopulation markers, including these specificity for M1-like macrophages (*IFN-γ, TNF-α, iNOS, IL-1β, CD68, Ccl2, Il-6, Ptgs2*) and M2-like macrophages (*CCL22, CSF2, VEGF, CD163, CD206, ARG1, Ccl24, Ccr2, Cd200r1, Chil3, Mrc1, Pparg*) were analyzed by qRT-PCR. In THP1 cell line-derived and RAW264.7 macrophages cultured with rhHMGB1 or rmHMGB1, M1-like related mRNA was significant upregulated but M2-like related mRNA was downregulated (Fig. 3C-D). However, in HMC3 cells, M1-like and M2-like mRNA were not changed (Fig. S5).

To explore whether HMGB1 secreted by GB cells upon TMZ treatment exerted function on TAMs through paracrine mechanisms, we knocked down HMGB1 with short hairpin RNA (shRNA) in a GB cell line LN229 and GL261 (Fig. S6). Co-culture of THP1 cell line-derived macrophages with sh*HMGB1*-LN229 cells with or without TMZ (500 μM) for 48 h significantly decreased M1-like activation associated genes, *CCL2, IL-1β, IL-6* and *TNF-α* (Fig. 3E). Then, we knocked down HMGB1 with shRNA in GL261 cells and generate orthotopic intracranial model in C57/BL6 mice to confirm the effect of HMGB1 on M1-like TAM polarization. Flow cytometry was used to explore TAMs isolated from the intracranial xenograft tumors under TMZ treatment. The results showed that the percentage of CD86^+^ cells (M1-like marker) elevated, but the percentage of CD86^+^ in CD45^+^ CD11b^+^ cells from xenograft tumors of GL261 cells with *Hmgb1* knockdown significantly reduced (Fig. 3F). Thus, GB-secreted HMGB1 upon TMZ treatment exerts a critical role in M1-like polarization of TAM.

### M1-like polarization of macrophages induced by HMGB1 enhances the sensitivity of GB to TMZ therapy

**Fig. 4:**
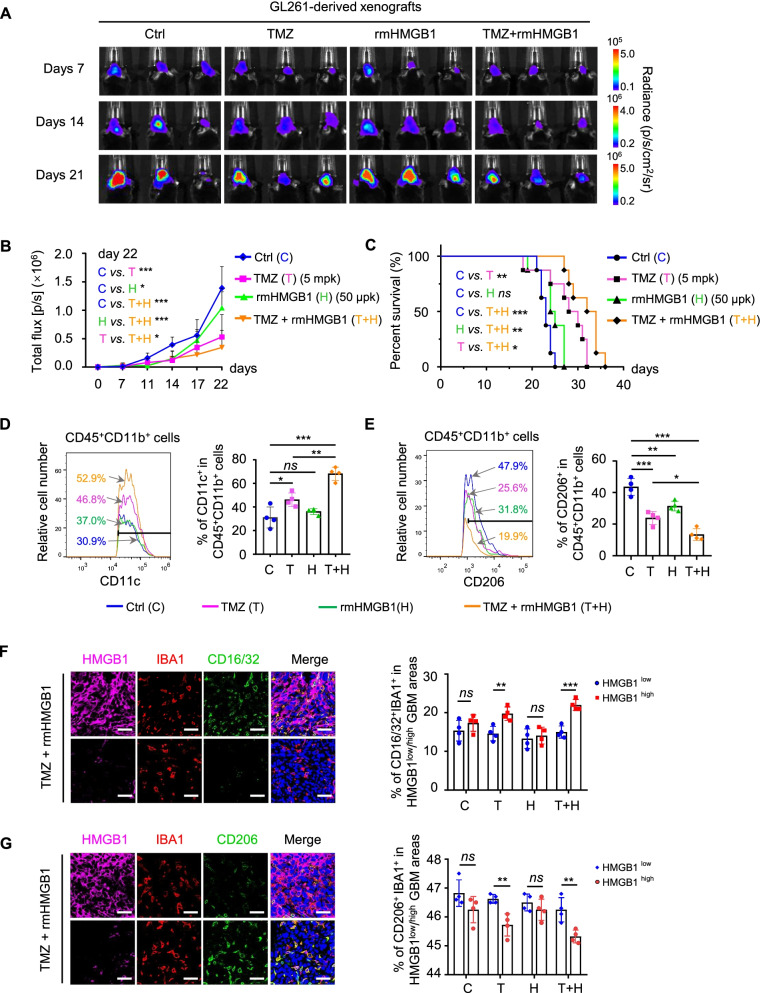
HMGB1 enhances the sensitivity of TMZ therapy by inducing M1-like polarization of TAMs. **A-B** Bioluminescent images (**A**) and quantification of tumors in mice (**B**) implanted with GL261 cells with treatment of Ctrl, TMZ, rmHMGB1 and TMZ plus rmHMGB1 for 7, 14 and 21 days. **C** The survival curves of tumor-bearing mice implanted with GL261 cells for indicated treatments. *n* = 8. **D** Representative flow cytometric analysis of tumor-infiltrating CD11c^+^ TAMs gated on live CD45^+^ CD11b^+^ TAMs isolated from GL261 cell-derived xenograft tumors with treatment of Ctrl (C), TMZ (T), rmHMGB1 (H) and TMZ plus rmHMGB1 (T + H) for 21 days (left panel). The histogram showed the statistical analysis (right panel). *n* = 4. **E** Representative flow cytometric analysis of tumor-infiltrating CD206^+^ TAMs gated on live CD45^+^ CD11b^+^ TAMs in GL261 cell-derived xenograft tumors with treatment of Ctrl (C), TMZ (T), rmHMGB1 (H) and TMZ plus rmHMGB1 (T + H) for 21 days (left panel). The histogram showed the statistical analysis (right panel). *n* = 4. **F** Representative IF of HMGB1 (purple), pan macrophage marker IBA1 (red) and M1-like TAM marker CD16/32 (green) in GL261 cell-derived xenograft tumors treated with TMZ plus rmHMGB1 (left panel). Quantitation of IBA1^+^/CD16/32^+^ TAM population in Ctrl, TMZ, rmHMGB1 and TMZ plus rmHMGB1 treatment groups (right panel). Scale bars = 50 μm. **G** Representative IF of HMGB1 (purple), IBA1 (red) and M2-like TAM marker CD206 (green) in GL261 cell-derived xenograft tumors treated with TMZ plus rmHMGB1 (left panel). Quantitation of IBA1^+^/CD206^+^ TAM population in Ctrl, TMZ, rmHMGB1 and TMZ plus rmHMGB1 treatment groups (right panel). Scale bars = 50 μm. *P < 0.05, **P < 0.01, ***P < 0.001, *ns* = no significance

Given the role of HMGB1 in the M1-like polarization of macrophages in vitro, we hypothesized that it may play a critical role in GB microenvironment to enhance anti-tumor immune responses. To analyze the effect of HMGB1 on GB progression, we implanted GL261 cells with luciferase intracranially into C57BL/6 mice, with administration of TMZ or rmHMGB1 or both. After eight days of tumor cell implantation, mice were treated with TMZ (5 mg/kg per day, i.p., for 5 consecutive days) together with or without rmHMGB1 (50 μg/kg per day, i.v., for 5 consecutive days). Tumor growth was monitored by bioluminescence using an In Vivo Imaging System (IVIS). Bioluminescent imaging indicated that combined treatment with rmHMGB1 significantly enhanced the anti-tumor effect of TMZ shown by markedly inhibited growth of GL261 cell-derived xenografts, while rmHMGB1 treatment alone exerted little benefits on the growth of GL261 cell-derived xenografts (Fig. 4A-B). Consequently, mice treated with TMZ and rmHMGB1 exhibited a significantly extended survival (Fig. 4C). In contrast, rmHMGB1 treatment alone had not effect on animal survival. Then, we used flow cytometry to analyze the proportion of M1-like (CD45^+^CD11b^+^CD11c^+^) and M2-like (CD45^+^CD11b^+^CD206^+^) TAM isolated from GL261 cell-derived xenografts. The results showed that the proportion of CD11c^+^ cells in the xenografts treated with TMZ or TMZ plus rmHMGB1 significantly increased (Fig. 4D). Meanwhile, a distinct reduction of the percentage of CD206^+^ cells was observed (Fig. 4E). Moreover, to determine extracellular HMGB1 distribution and its correlation with the subtype of TAMs in GB xenografts, frozen sections were stained for HMGB1 together with pan TAM marker IBA1 and the M1/M2-like TAM marker (M1: CD16/32; M2: CD206) in GL261 cell-derived xenografts. We found that in HMGB1^high^ region of GL261 cell-derived xenografts, M1-like TAMs markedly increased with significantly reduced M2-like TAMs (Fig. 4F-G and Fig. S7A-B). These data demonstrate that HMGB1 secreted by GB cells enhanced the sensitivity of the tumors to TMZ therapy by inducing M1-like polarization of macrophages. In addition, immunofluorescent staining showed that administration of TMZ plus rmHMGB1 reduced Ki67-postive proliferative cells and increased the number of apoptotic cells as marked by cleaved-caspase-3 in GL261 cell-derived xenografts (Fig. S8A-B). We further found that GB sections with high extracellular HMGB1 showed enhanced cleaved-caspase-3 staining in patients (GB0436 and GB8421) with TMZ treatment (Fig. S8C). These data demonstrate that HMGB1 secreted by GB upon TMZ treatment is a critical paracrine factor to mediate the tumor-suppressive effect of M1-like TAMs to restrain GB growth.

### RAGE is a receptor for HMGB1 on TAMs

**Fig. 5:**
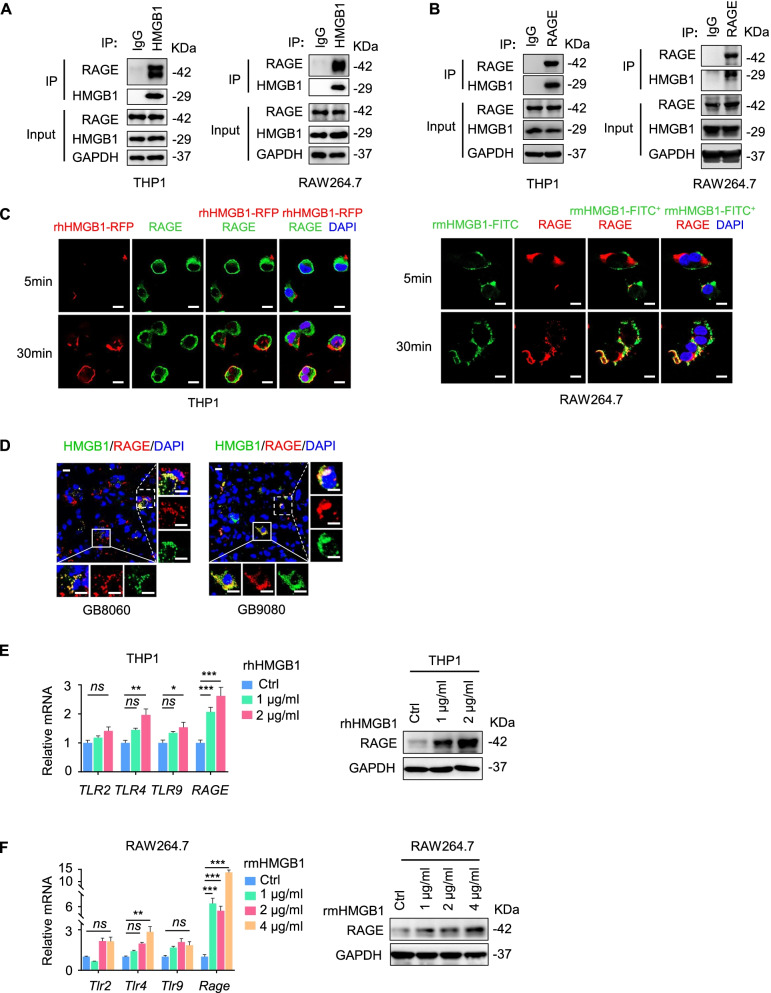
HMGB1 binds to RAGE on TAMs. **A** Co-IP assays of interaction of HMGB1 with RAGE in THP1 cell line-derived macrophages (left panel) and RAW264.7 macrophages (right panel). Cell lysates were immunoprecipitated with anti-HMGB1 antibody, then immunoblotted with anti-HMGB1 and anti-RAGE antibodies. **B** Co-IP assays of interaction of RAGE with HMGB1 in THP1 cell line-derived macrophages (left panel) and RAW264.7 macrophages (right panel). Cell lysates were immunoprecipitated with anti-RAGE antibody, then immunoblotted with anti-HMGB1 and anti-RAGE antibodies. **C** THP1 cell line-derived macrophages (left panel) and RAW264.7 macrophages (right panel) were treated with RFP-labeled rhHMGB1 or FITC-labeled rmHMGB1 for 5 and 30 min, respectively. Scale bars = 10 μm. **D** Colocalization of HMGB1 (green) and RAGE (red) in human GB samples (GB8060 and GB9080) with TMZ treatment. Scale bars = 10 μm. **E** The mRNA expression of HMGB1 receptors (*TLR2, TLR4, TLR9, RAGE*) by THP1 cell line-derived macrophages after stimulation with rhHMGB1 at indicated concentrations for 24 h (left panel). Immunoblot of RAGE in THP1 cell line-derived macrophages with or without rhHMGB1 treatment for 48 h (right panel). **F** The mRNA expression of HMGB1 receptors (*Tlr2, Tlr4, Tlr9, Rage*) by RAW264.7 macrophages after stimulation with rmHMGB1 at indicated concentrations for 24 h (left panel). Immunoblot of RAGE in RAW264.7 macrophages with or without rmHMGB1 treatment for 48 h (right panel). *P < 0.05, **P < 0.01, ***P < 0.001,* ns* = no significance

To explore the molecular mechanisms underlying M1-like polarization of macrophages promoted by HMGB1, we used co-immunoprecipitation (Co-IP) to examine the receptor interacting with HMGB1 expressed by macrophages. We demonstrated that RAGE showed a high affinity for HMGB1 in macrophages (Fig. 5A-B). To further confirm the specificity of RAGE as a receptor for HMGB1 expressed by macrophages, we used RFP or FITC labeled-rhHMGB1/rmHMGB1 to test its binding to macrophages. We found that the binding of rHMGB1 with RAGE on macrophages increased with the exposure time (Fig. 5C). Moreover, immunofluorescence showed co-expression of HMGB1 and RAGE on human GB samples (GB8060 and GB9080) with TMZ treatment (Fig. 5D). We also observed that the mRNA and protein levels of RAGE markedly increased with no change in the levels of other putative receptors on THP1 cell line-derived macrophages and RAW264.7 macrophages after stimulation with rhHMGB1 or rmHMGB1 (Fig. 5E-F). These data indicate that RAGE is a major receptor on macrophages serving paracrine signaling in response to HMGB1 produced by GB cells upon TMZ treatment.

### The RAGE-NFκB-NLRP3 inflammasome pathway is involved in M1-like polarization of macrophages via HMGB1

**Fig. 6:**
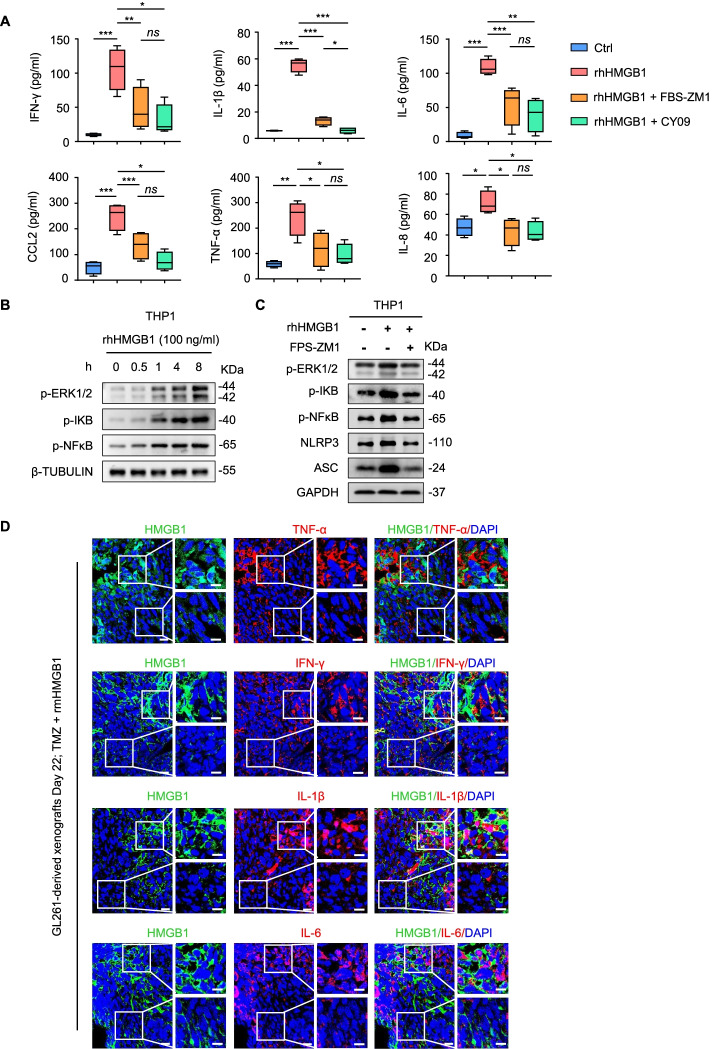
HMGB1 enhances M1-like polarization of macrophages by activating ERK1/2-NFκB-NLRP3 inflammasome pathway. **A** Cytokines released into supernatants of THP1 cell line -derived macrophages, including IFN-γ, IL-1β, IL-6, IL-8, CCL2 and TNF-α were detected by ELISA after indicated treatment for 24 h. rhHMGB1: 1 μg/ml; FBS-ZM1: 100 nM; CY-09: 5 μM. **B** Immunoblot of p-ERK1/2, p-IKB and p-NFκB in lysates of THP1 cell line-derived macrophages treated with rhHMGB1 (100 ng/ml) for indicated times. **C** THP1 cell line-derived macrophages were pre-treated with FBS-ZM1 (100 nM) for 2 h, then stimulated with rhHMGB1 (1 μg/ml) for 24 h. p-ERK1/2, p-IKB, p-NFκB, NLRP3 and ASC levels were detected by immunoblot. **D** Representative IF of the cytokines (M1-like: TNF-α, IFN-γ, IL-1β, IL-6) (red) and HMGB1 (green) in GL261 cell-derived xenograft tumors treated with TMZ plus rmHMGB1. Squares are enlarged and shown on the right side of each image. Scale bars = 25 μm. *P < 0.05, **P < 0.01, ***P < 0.001, *ns* = no significance

HMGB1 polarizes a M1-like phenotype of macrophages by promoting the release of pro-inflammatory cytokines through activation of inflammasomes [42, 43]. We found significantly increased levels of TNF-α, IFN-γ, IL-1β, IL-6, IL-8 and CCL2 in the supernatants released by THP1 cell line-derived macrophages cultured with rhHMGB1. The effect of rhHMGB1 was reduced by addition of FPS-ZM1, an inhibitor of RAGE (Fig. 6A). After culture with CY-09, an inhibitor of NLRP3 which is a key component of the protein complex in inflammasome, the levels of pro-inflammatory cytokines produced by THP1 cell line-derived macrophages upon HMGB1 also significantly decreased (Fig. 6A). These results suggest that HMGB1 promotes the release of pro-inflammatory cytokines by macrophages through RAGE and inflammasome. In macrophages, HMGB1 activates RAGE through the phosphorylation of the extracellular regulated protein kinases (ERK1/2) followed by the activation of NFκB and cytokine production [17, 19, 38]. We found that rhHMGB1 increased the phosphorylation of ERK1/2, IKB and NFκB in THP1 cell line-derived macrophages (Fig. 6B), which was markedly suppressed by addition of FPS-ZM1 (Fig. 6C), indicating that RAGE activation by HMGB1 is linked to the phosphorylation of ERK1/2 and IKB that activates NFκB to release pro-inflammatory cytokines by macrophages through NLRP3-dependent inflammasomes. To better exhibit the direct proof in vivo, we stained the sections from GL261 cell-derived xenografts treated with TMZ plus rmHMGB1, and found that the tumor regions with obvious extracellular HMGB1 highly expressed TNF-α, IFN-γ, IL-1β, IL-6, IL-8 and CCL2 detected by immunofluorescence, which are cytokines following the activation of RAGE-NFκB-NLRP3 inflammasome (Fig. 6D and Fig. S9A). Furthermore, our data showed that extracellular HMGB1 in xenografts induced the production of NLRP3 and ASC, the key component in inflammasome (Fig. S9B), which simultaneously promoted the release of TNF-α, IFN-γ, IL-1β, IL-6, IL-8 and CCL2 in an NLRP3 inflammasome-dependent manner (Fig. S10). Collectively, the results confirmed the pathway of RAGE-NFκB-NLRP3 inflammasome via HMGB1 to induce M1-like polarization of TAMs in GB.

## Discussion

Autophagy induction is one of the hallmark effects of anti-GB therapy with temozolomide (TMZ). Understanding the mechanisms of autophagy under TMZ treatment is crucial since chloroquine (CQ) and hydroxychloroquine (HCQ), which disrupt lysosomal acidification and manifest anti-cancer activity in mice, have not improved overall survival of GB patients when combined with TMZ [5].

Recent studies have demonstrated that autophagy not only controls protein degradation but also protein secretion known as secretory autophagy [8]. In this study, we demonstrated, for the first time, that extracellular HMGB1 released through secretory autophagy acts as a regulator of the crosstalk between GB cells and immune microenvironment that play major roles in tumor sensitivity towards TMZ. HMGB1 induces M1-like polarization of TAMs and promotes the secretion of proinflammatory cytokines, which trigger potent antitumor immunity [44] and converts an immunological “cold” microenvironment, which is frequently present in GB, to an immunological “hot” one [45, 46].

Inhibition of autophagosome formation via LY294002 or 3-methyladenine (3-MA) in GB cells treated with TMZ decreased extracellular HMGB1, which highlighted the role of autophagy promoted by TMZ treatment. ATG5-mediated classical autophagy pathway and GORASP2, crucial for the unconventional secretion of cytoplasmic proteins [8, 47], have been reported to promote HMGB1 release by 293 T cells under starvation or lipopolysaccharide (LPS) treatment [8, 48]. Deficiency of ATG5 or GORASP2 in GB cells significantly reduced HMGB1 secretion during TMZ treatment, confirming that HMGB1 is contained in autophagosomes of GB cells.

Intracellular HMGB1 is stored in the nucleus as a DNA chaperone and loss of HMGB1 increases DNA damage and decreases DNA repair efficiency in response to chemotherapy [49]. Analysis of cancer genome databases revealed an inverse relationship between HMGB1 mRNA levels and overall survival of GB patients. Correlation between HMGB1 and TMZ treatment has been supported by measurement of HMGB1 expression levels via IHC and ELISA from paired GB patient samples. Under TMZ treatment, GB samples with lower HMGB1 IHC score in nuclei showed higher levels in sera. Higher HMGB1 levels in sera of GB patients favored overall survival indicating the increased sensitivity to TMZ. Culture of GB cells with rhHMGB1 in vitro did not show significant effect on the proliferation of GB cells (Fig. S11A). We also cultured GB cells with rhHMGB1 and TMZ in vitro, with no effect on the sensitivity of tumor cells to TMZ (Fig. S11B). Thus, extracellular HMGB1 secreted by GB cells under TMZ treatment have little effect on tumor cells.

Accumulating evidence shows that secreted proteins are responsible for the crosstalk among cells in the TME, which may play a critical role in the regulation of drug responses [50]. A recent study showed that HMGB1 acted as a ligand for toll-like receptor 2 (TLR2) on bone marrow-derived GB-infiltrating dendritic cells to elicit tumor regression [22]. TME of GB is composed of multiple components, including parenchymal cells, soluble factors, blood vessels, extracellular matrix and infiltrating immune cells in which TAMs constitute a dominant cell population [51, 52]. Our study revealed that RAGE, the receptor for HMGB1, is mainly located in TAMs and is responsible for the crosstalk with HMGB1 in the GB microenvironment to regulate TMZ responses. It is well recognized that TAMs include two major populations: tumor-supportive M2-like macrophages and tumor-suppressive M1-like macrophages. In our study, we revealed that HMGB1 induced M1-like polarization of TAMs in TME to activate ERK1/2-NFκB-NLRP3 inflammasome pathway resulting in the release of cytokines IFN-γ, IL-1β, IL-6, CCL2, TNF-α and IL-8. Knockdown of HMGB1 in GB cells abolished M1-like polarizations of TAMs in co-culture system upon TMZ treatment. HMGB1 in vivo significantly increased polarization of tumor-suppressive TAMs (M1-like) and inhibited intracranial GB growth. Therefore, both GB-secreted HMGB1 via secretory autophagy and TAMs constitute a paracrine signaling loop in TME which is critical for systemic antitumor immune responses.

It was previously reported that the polarization of M1-like macrophages is characterized by NFκB-NLRP3 activation [53, 54] and release of proinflammatory mediators including IFN-γ, IL-1β, IL-6, CCL2, TNF-α, and IL-8 [43, 55]. Our study assessed ERK1/2, IKB and NFκB-NLRP3 pathway and found that these molecules were activated in macrophages by HMGB1. Proinflammatory cytokines are elevated after stimulation of macrophages by rHMGB1 which signals through RAGE [56].

**Fig. 7:**
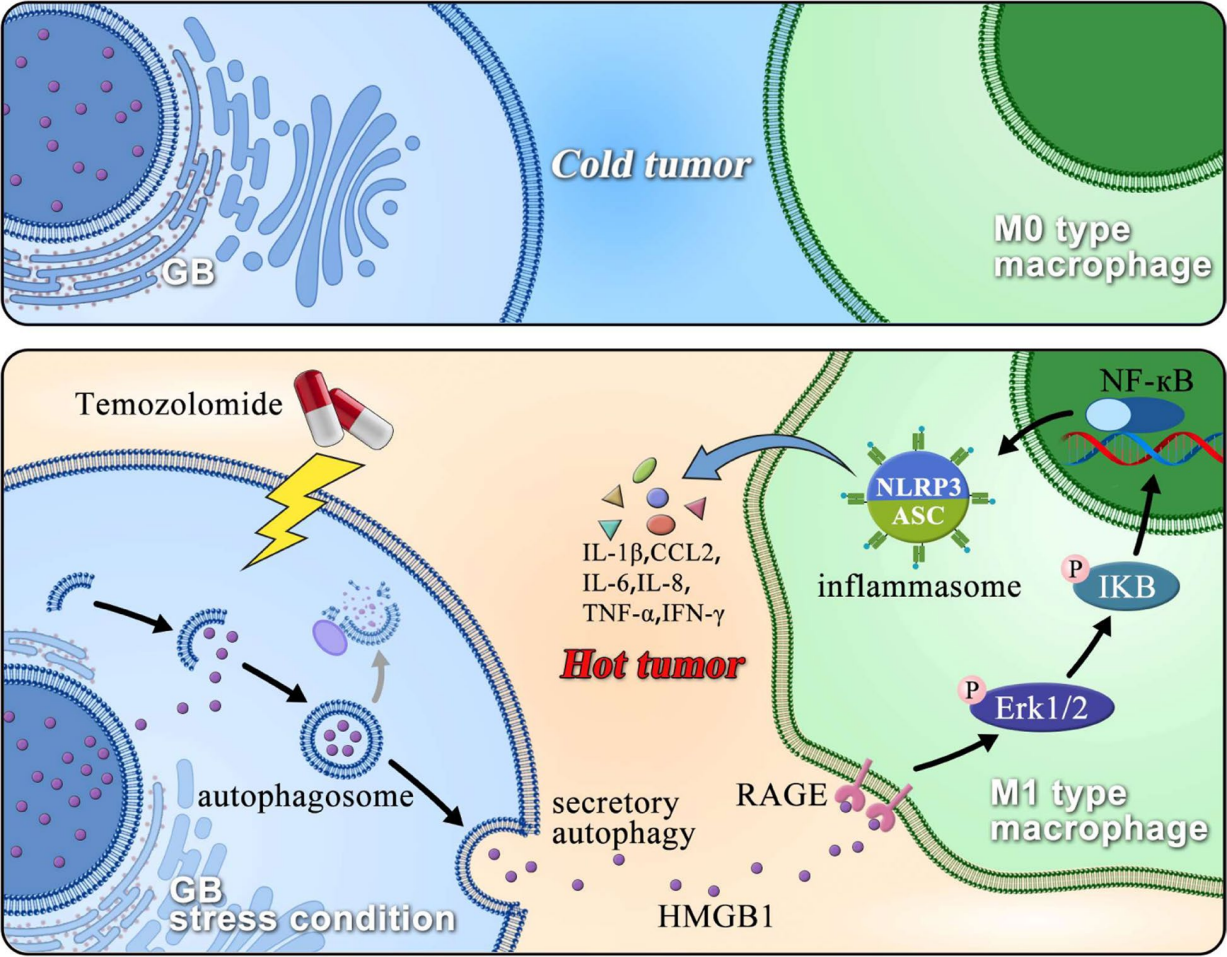
A working model for the HMGB1 and GB chemosensitivity. When GBs are treated with TMZ chemotherapy, secretory autophagy is activated to release HMGB1 which promotes M1-like polarization of TAMs and TMZ chemosensitivity of GB

In summary, we demonstrated that enhanced secretory autophagy in GB under TMZ treatment facilitated M1-like polarization of TAMs to inhibit GB growth. HMGB1 acted as a key regulator in mediating the crosstalk between GB cells and tumor-suppressive M1-like TAMs in the TME with the presence of the chemotherapeutic agent TMZ (Fig. 7). This may explain at least in part the reason why autophagy inhibition in combination with TMZ failed to cause effective tumor regression. Our study suggests a novel approach to enhancing the chemosensitivity of GB by targeting the metabolic pathway of HMGB1 in TME.

## Conclusions

Our results indicate that enhanced secretory autophagy in GB with TMZ treatment is involved in the exocytosis of HMGB1. HMGB1 acts as a key regulator in the crosstalk between GB cells and tumor-suppressive M1-like TAMs in GB microenvironment and enhances TMZ sensitivity of GB cells. Thus, HMGB1 may be considered as an adjuvant for the chemotherapeutic agent TMZ.

## Supplementary Information

**Additional file 1: Figure S1.** The levels of secretory autophagy proteins in the supernatants of GB cells upon TMZ treatment.

**Additional file 2: Figure S2.** Involvement of secretory autophagy in the release of HMGB1 in GB upon TMZ treatment.

**Additional file 3: Figure S3.** Release of HMGB1 by TMZ-treated GB cells dependent on the formation of autophagic vacuoles.

**Additional file 4: Figure S4.** The correlation between mRNA HMGB1 and the prognosis of GB patients from database.

**Additional file 5: Figure S5.** HMGB1 has no effect on microglia HMC3.

**Additional file 6: Figure S6.** Knockdown of HMGB1 in LN229 cells and GL261 cells.

**Additional file 7: Figure S7.** The expression of M1/M2-like TAM markers in GL261 cell-derived xenograft tumors.

**Additional file 8: Figure S8.** The expression of apoptotic proteins in extracellular HMGB1-enriched regions of GL261 cell-derived xenograft tumors.

**Additional file 9: Figure S9.** The expression of cytokines released by the activation of RAGE-NFκB-NLRP3 inflammasome pathway in extracellular HMGB1-enriched regions of GL261 cell-derived xenograft tumors.

**Additional file 10: Figure S10.** The expression of cytokines released by the activation of RAGE-NFκB-NLRP3 inflammasome pathway in extracellular NLRP3-enriched regions of GL261 cell-derived xenograft tumors.

**Additional file 11: Figure S11.** HMGB1 has no effect on GB cell proliferation *in vitro*.

**Additional file 12: Table 1.** Clinicopathological characteristics of human GBs used in this study. **Table 2.** Primers used for qRT-PCR. **Table 3.** The sequences of shRNA.

## Data Availability

All data used in this study are included within the article and additional files.

## Abbreviations

GB: Glioblastoma
GBM: Glioblastoma multiforme
TMZ: Temozolomide
HMGB1: High mobility group box 1
TEM: Transmission electron microscopy
ELISA: Enzyme-linked immunosorbent assay
qRT-PCR: Quantitative reverse transcription polymerase chain reaction
GFP: Green fluorescent protein
IHC: Immunohistochemistry
TME: Tumor microenvironment
TCGA: The cancer genome atlas
CGGA: The Chinese glioma genome atlas
LC3B: Microtubule-associated protein 1 light chain
ATG5: Autophagy-related 5
ATG7: Autophagy-related 7
BECN1: Beclin 1
SQSTM1: Sequestosome 1
STX17: Syntaxin 17
TLR2: Toll-like receptor 2
TLR4: Toll-like receptor 4
TLR9: Toll-like receptor 9
RAGE: Receptor for advanced glycation end products
ERK1/2: Extracellular signal-regulated protein kinases 1 and 2
IKB: Inhibitor of kappa-b
LPS: Lipopolysaccharide
DMSO: Dimethyl sulfoxide
NLRP3: NLR family pyrin domain containing 3
TAMs: Tumor-associated macrophages
CQ: Chloroquine
HCQ: Hydroxychloroquine
GORASP2: Golgi reassembly stacking protein 2
