# Supplementary figures and images for: Autophagy-based unconventional secretion of HMGB1 in glioblastoma promotes chemosensitivity to temozolomide through macrophage M1-like polarization
Source: J Exp Clin Cancer Res. 2022 Feb 22;41:74. doi: 10.1186/s13046-022-02291-8 (PMC8862393; doi:10.1186/s13046-022-02291-8)

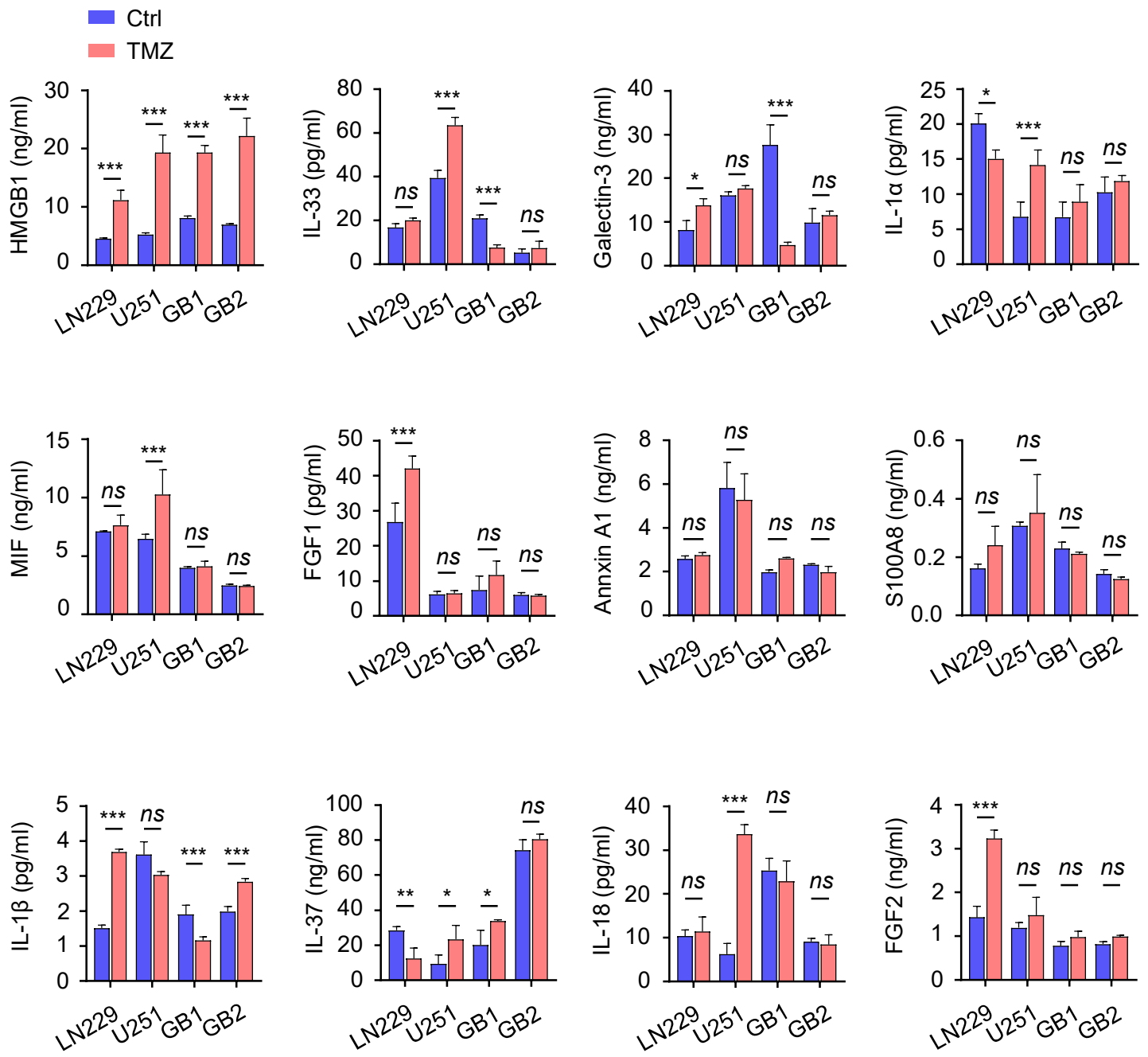

Figure S1

Supplement: Supplementary file 1 — Additional file 1: Figure S1. The levels of secretory autophagy proteins in the supernatants of GB cells upon TMZ treatment. [file 13046_2022_2291_MOESM1_ESM.pdf]

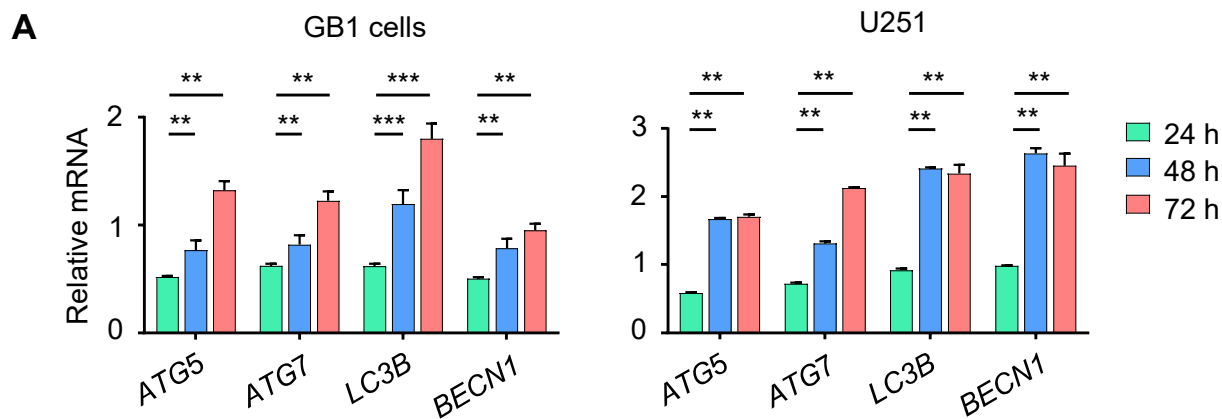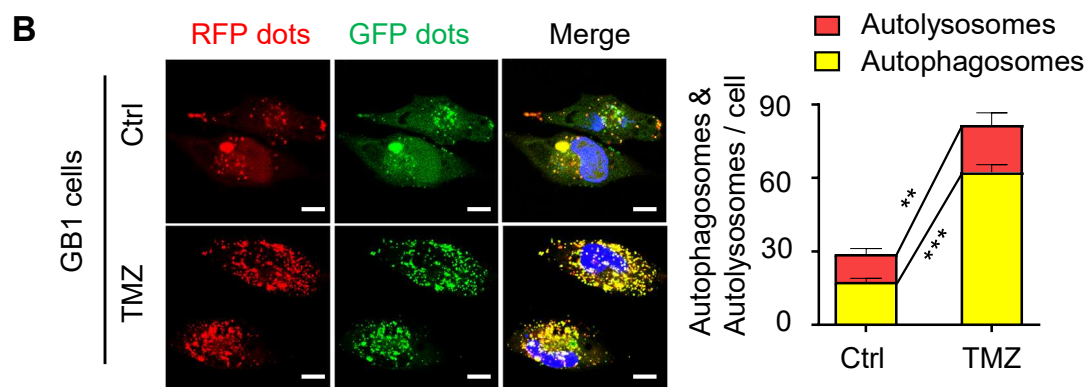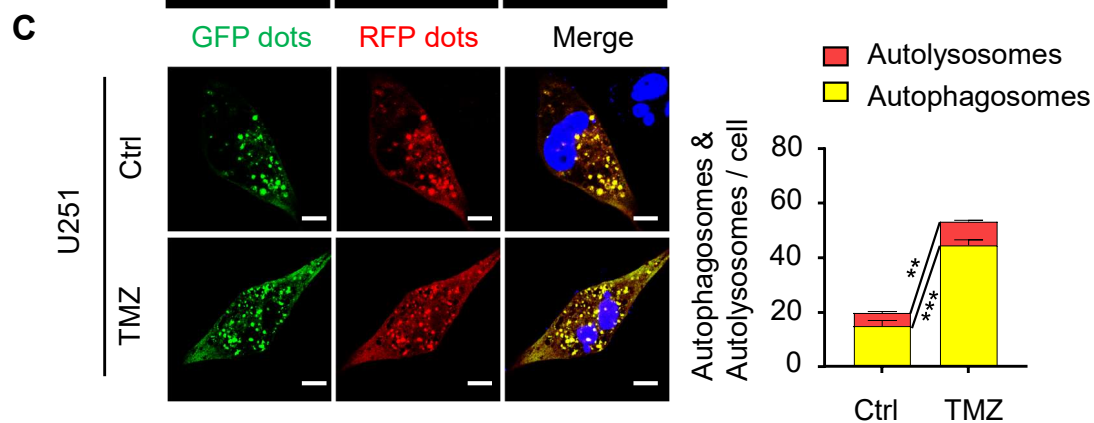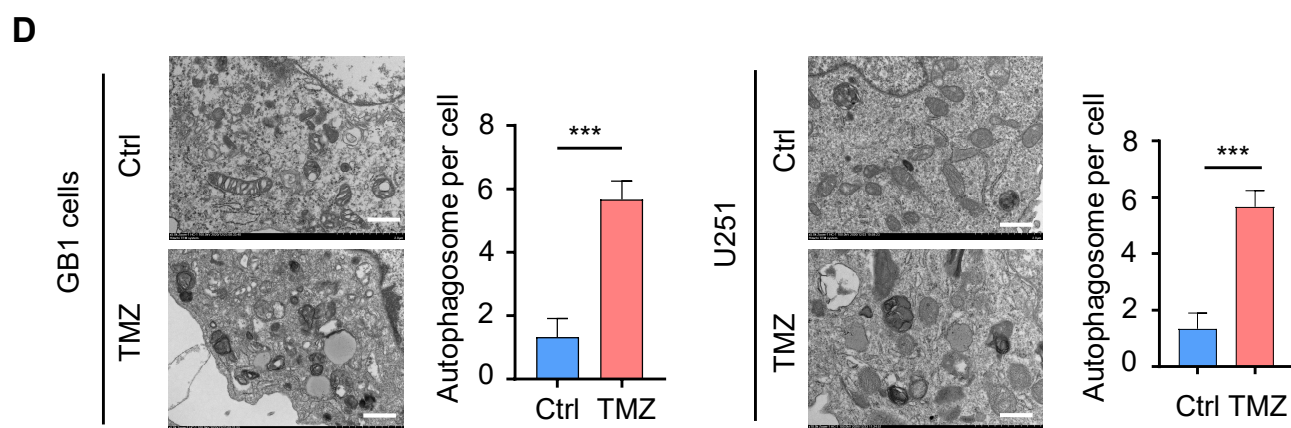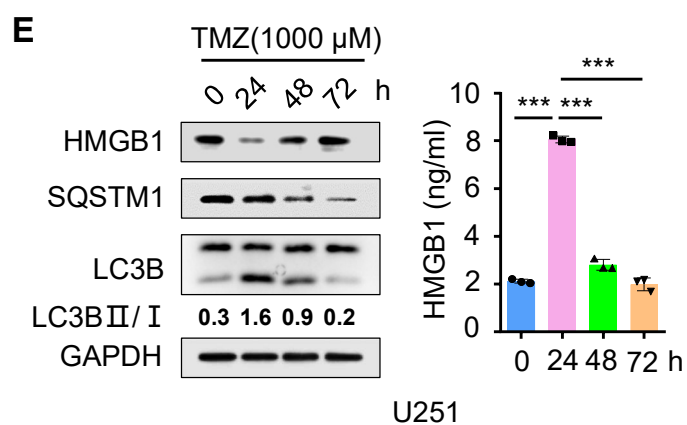

Figure S2

Supplement: Supplementary file 2 — Additional file 2: Figure S2. Involvement of secretory autophagy in the release of HMGB1 in GB upon TMZ treatment. [file 13046_2022_2291_MOESM2_ESM.pdf]

**A**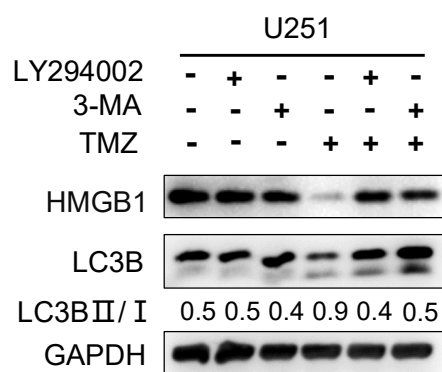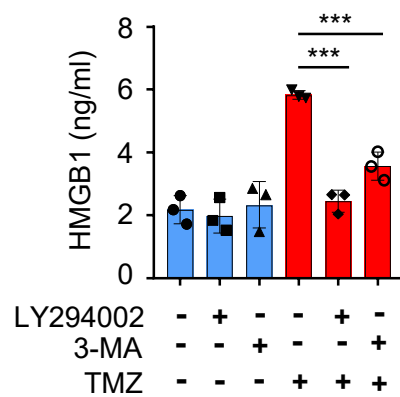**B**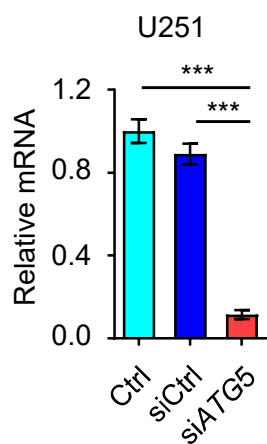**C**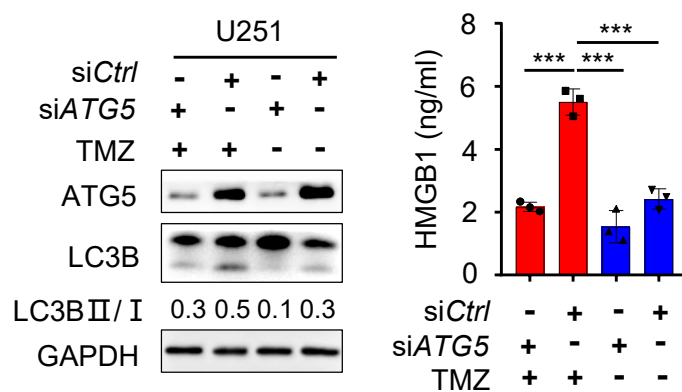**D**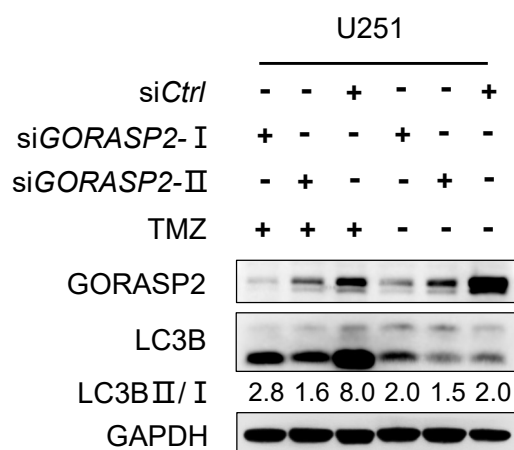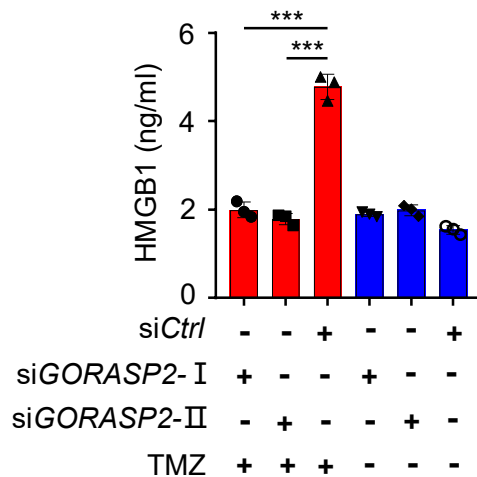

Figure S3

Supplement: Supplementary file 3 — Additional file 3: Figure S3. Release of HMGB1 by TMZ-treated GB cells dependent on the formation of autophagic vacuoles. [file 13046_2022_2291_MOESM3_ESM.pdf]

**A**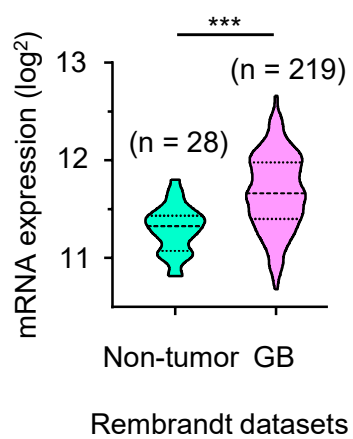**B**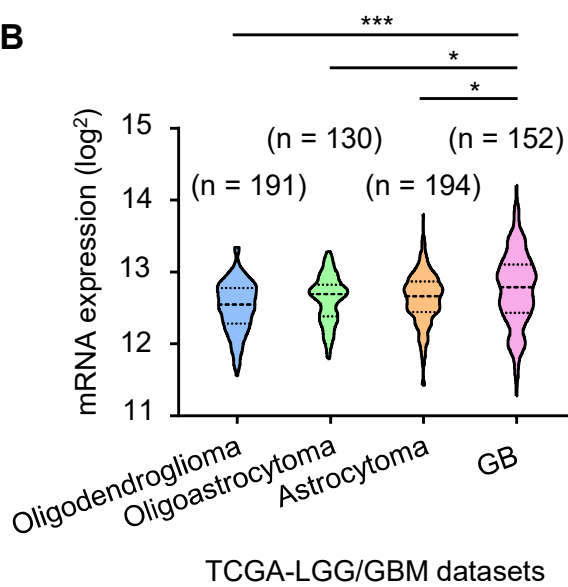

Figure S4

Supplement: Supplementary file 4 — Additional file 4: Figure S4. The correlation between mRNA HMGB1 and the prognosis of GB patients from database. [file 13046_2022_2291_MOESM4_ESM.pdf]

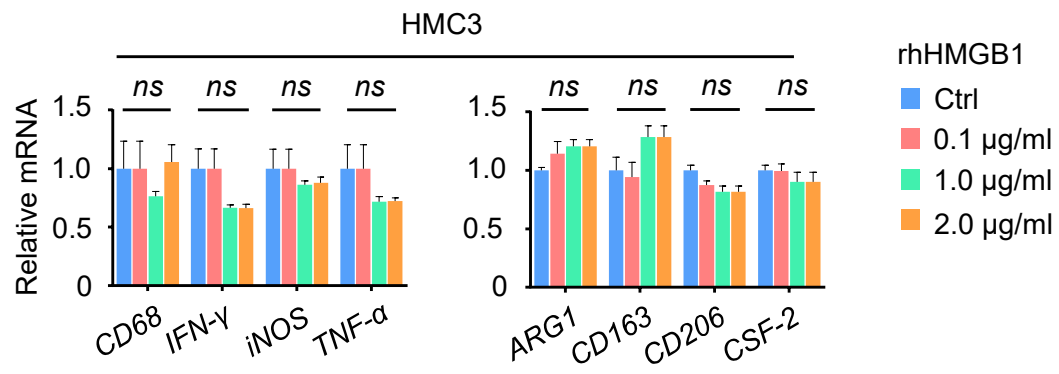

Figure S5

Supplement: Supplementary file 5 — Additional file 5: Figure S5. HMGB1 has no effect on microglia HMC3. [file 13046_2022_2291_MOESM5_ESM.pdf]

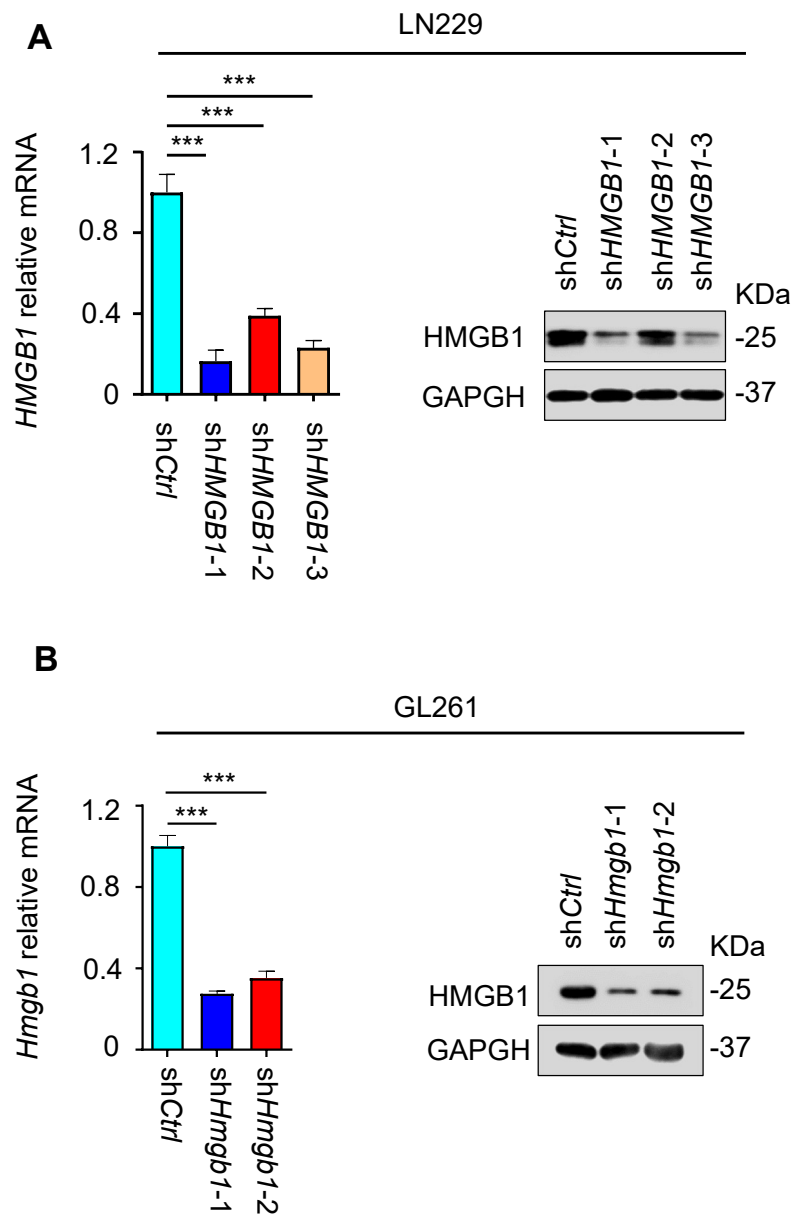

Figure S6

Supplement: Supplementary file 6 — Additional file 6: Figure S6. Knockdown of HMGB1 in LN229 cells and GL261 cells. [file 13046_2022_2291_MOESM6_ESM.pdf]

**A**

GL261-derived xenografts Day 22

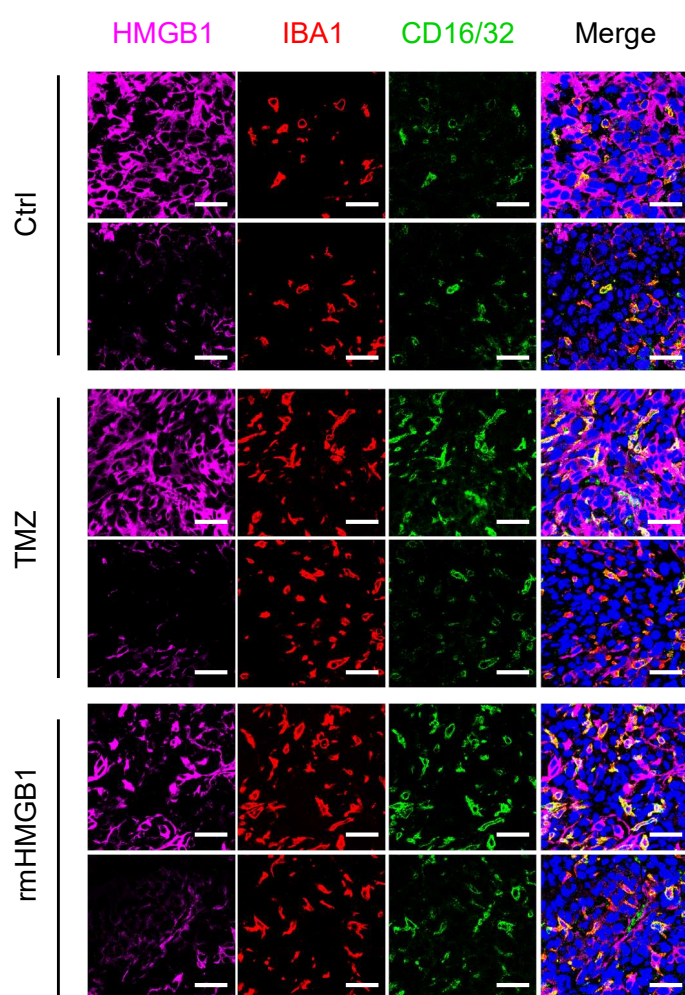**B**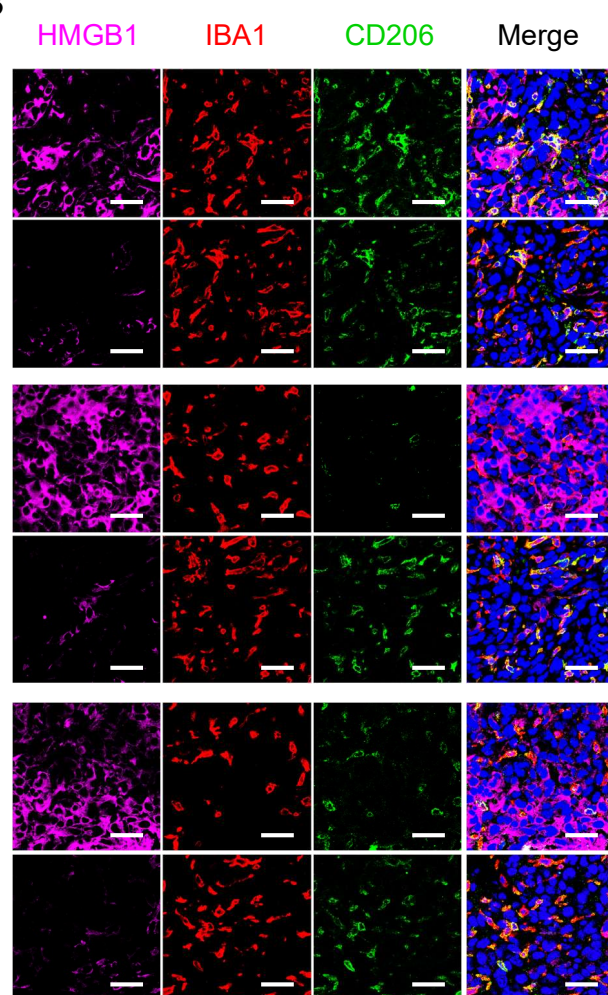

Figure S7

Supplement: Supplementary file 7 — Additional file 7: Figure S7. The expression of M1/M2-like TAM markers in GL261 cell-derived xenograft tumors. [file 13046_2022_2291_MOESM7_ESM.pdf]

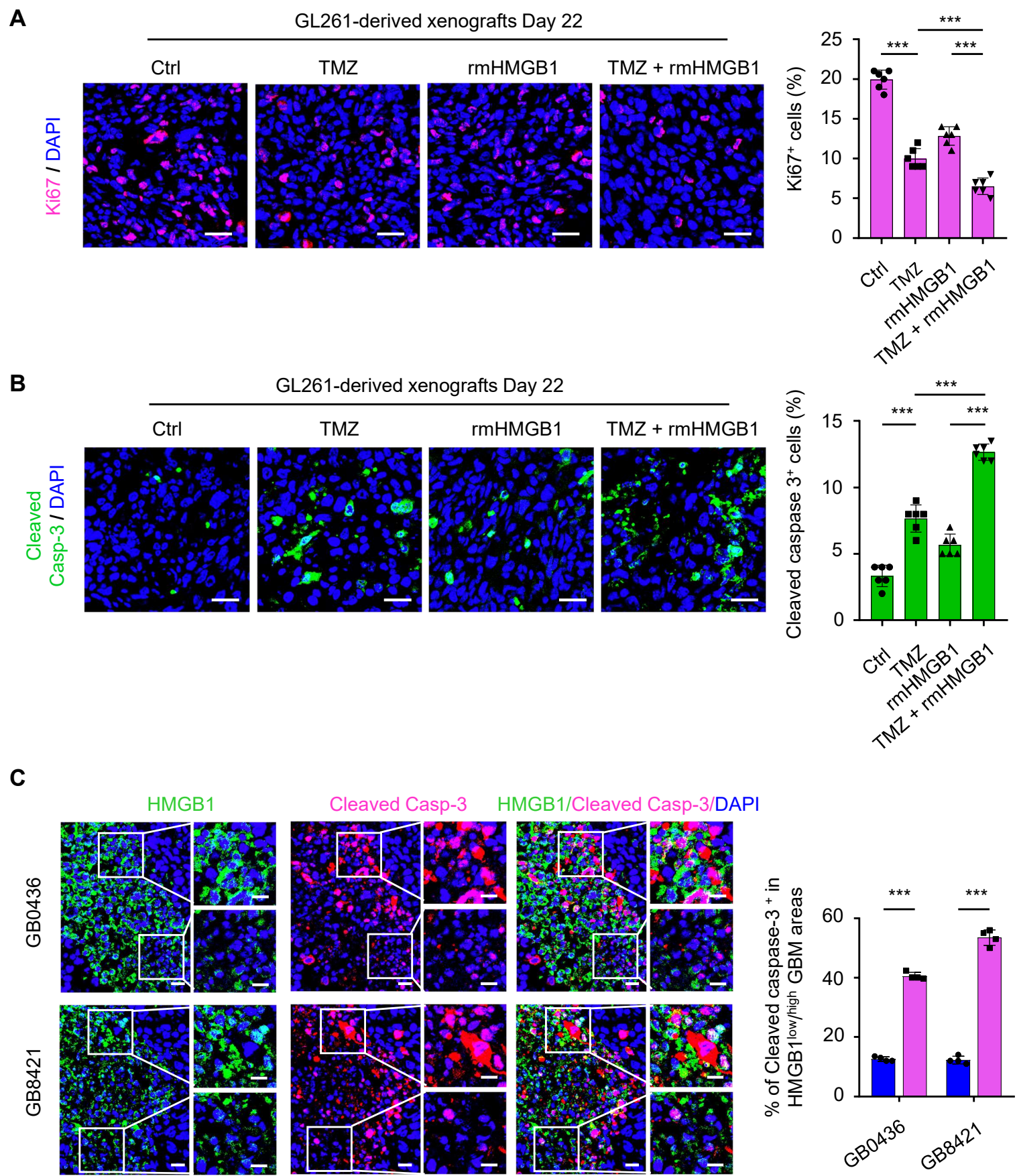

Figure S8

Supplement: Supplementary file 8 — Additional file 8: Figure S8. The expression of apoptotic proteins in extracellular HMGB1-enriched regions of GL261 cell-derived xenograft tumors. [file 13046_2022_2291_MOESM8_ESM.pdf]

**A**

GL261-derived xenografts Day 22  
TMZ + rmHMGB1

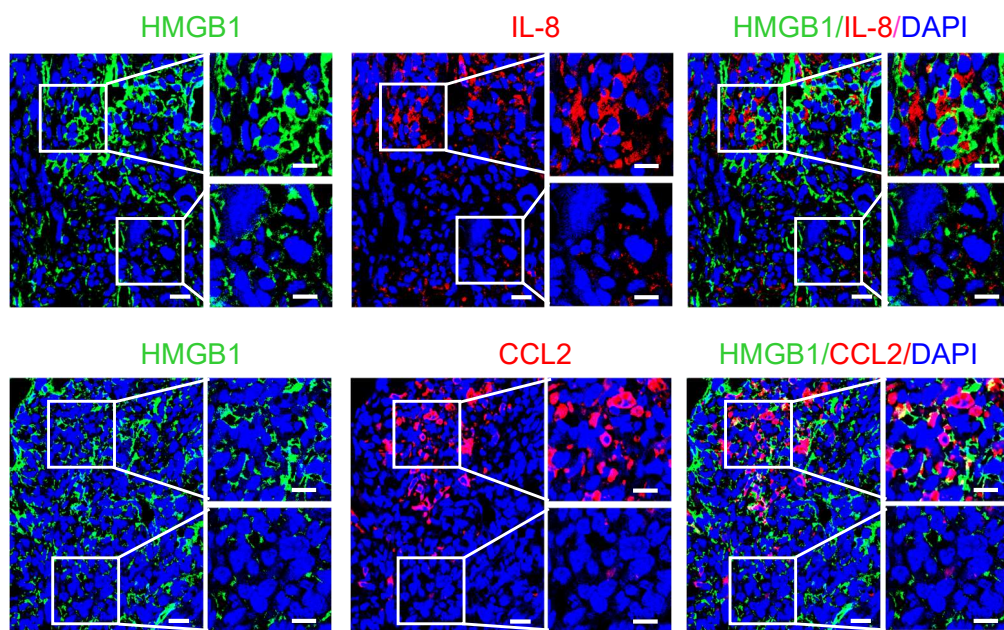**B**

GL261-derived xenografts Day 22  
TMZ + rmHMGB1

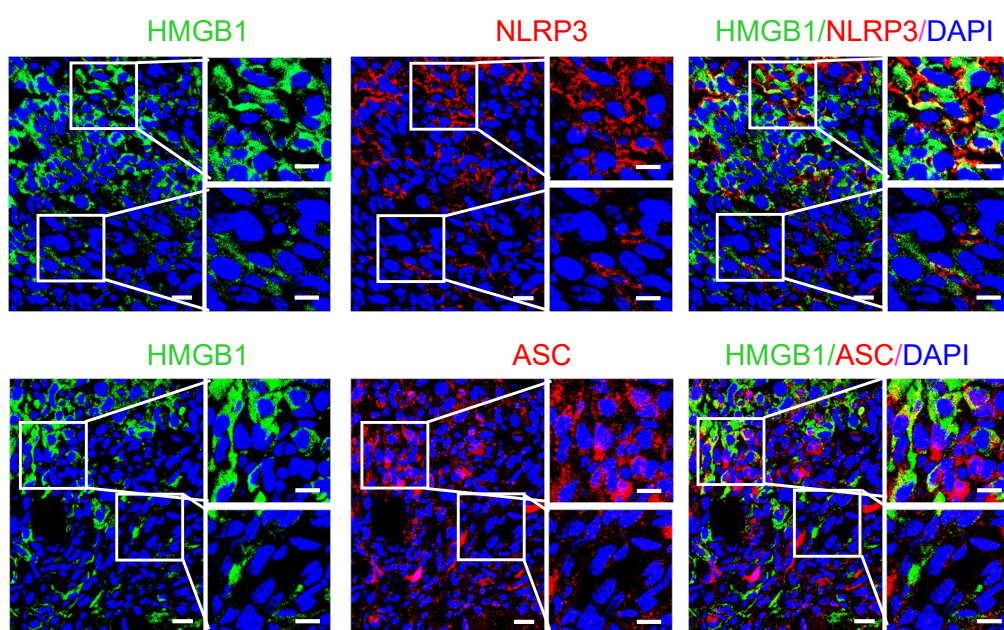

Figure S9

Supplement: Supplementary file 9 — Additional file 9: Figure S9. The expression of cytokines released by the activation of RAGE-NFκB-NLRP3 inflammasome pathway in extracellular HMGB1-enriched regions of GL261 cell-derived xenograft tumors. [file 13046_2022_2291_MOESM9_ESM.pdf]

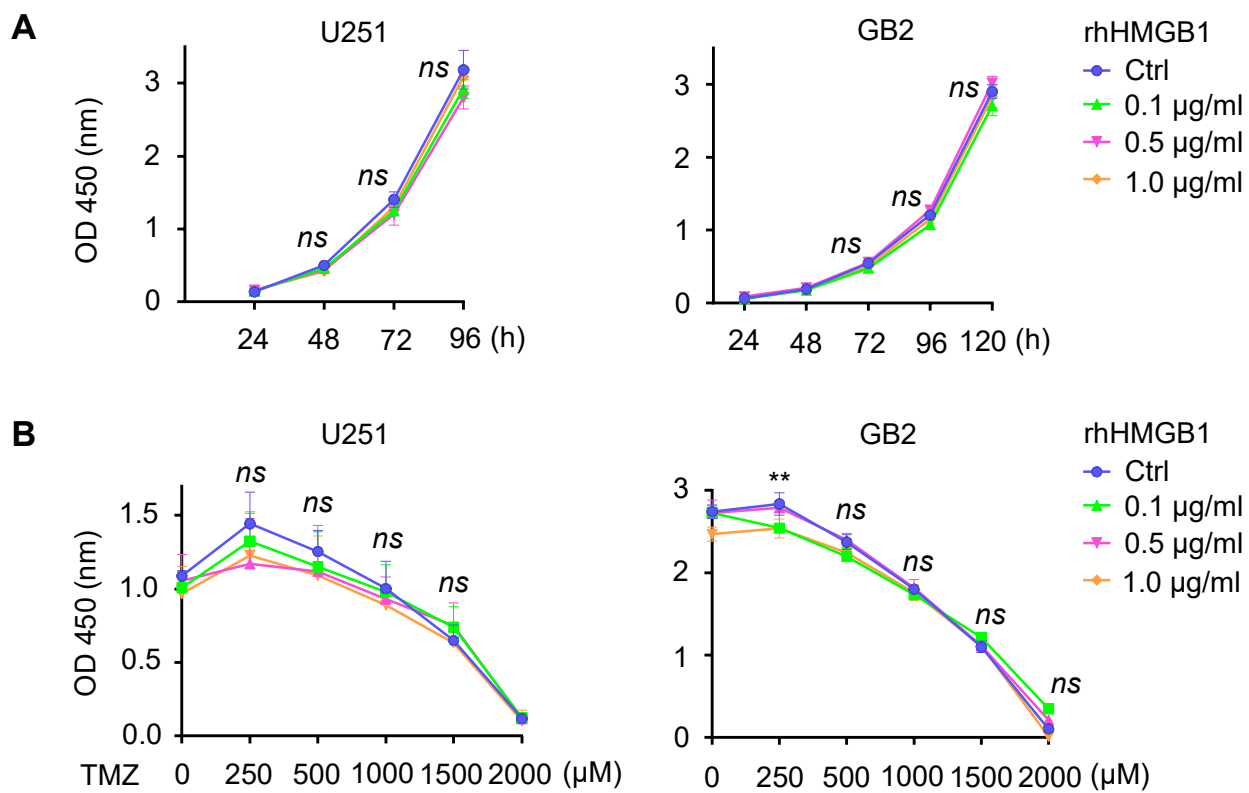

Figure S11

Supplement: Supplementary file 11 — Additional file 11: Figure S11. HMGB1 has no effect on GB cell proliferation in vitro. [file 13046_2022_2291_MOESM11_ESM.pdf]
