# Supplementary material for: Autophagy-based unconventional secretion of HMGB1 in glioblastoma promotes chemosensitivity to temozolomide through macrophage M1-like polarization
Source: J Exp Clin Cancer Res. 2022 Feb 22;41:74. doi: 10.1186/s13046-022-02291-8 (PMC8862393; doi:10.1186/s13046-022-02291-8)
Supplement: Supplementary file 12 — Additional file 12: Table 1. Clinicopathological characteristics of human GBs used in this study. Table 2. Primers used for qRT-PCR. Table 3. The sequences of shRNA. [file 13046_2022_2291_MOESM12_ESM.docx]

**Supplementary Table 1** Clinicopathological characteristics of human GBs used in this study.

| Number | Specimen | Histopathology | WHO Grade | Gender | Age | Tumor location | Extent of surgical  resection | TMZ treatment | Recurrent |
| --- | --- | --- | --- | --- | --- | --- | --- | --- | --- |
| 1 | GB6429 | GB | Ⅳ | Male | 39 | Right frontal-parietal | GTR | No | No |
| 2 | GB7225 | GB | Ⅳ | Male | 49 | Right parietal | GTR | Yes | Yes |
| 3 | GB1388 | GB | Ⅳ | Male | 55 | Left temporal | PR | Yes | Yes |
| 4 | GB8060 | GB | Ⅳ | Male | 48 | Right frontal | GTR | Yes | Yes |
| 5 | GB9080 | GB | Ⅳ | Female | 52 | Right parietal-occipital | GTR | Yes | Yes |
| 6 | GB0436 | GB | Ⅳ | Male | 39 | Right temporal-parietal | GTR | Yes | Yes |
| 7 | GB8421 | GB | Ⅳ | Male | 57 | Right temporal | GTR | Yes | Yes |
| 8 | GB0055 | GB | Ⅳ | Female | 60 | Right temporal | PR | Yes | Yes |
| 9 | GB5432 | GB | Ⅳ | Male | 37 | Right temporal | GTR | Yes | Yes |
| 10 | GB9205 | GB | Ⅳ | Female | 47 | Right temporal | GTR | Yes | Yes |
| 11 | GB9243 | GB | Ⅳ | Female | 38 | Right temporal | GTR | Yes | Yes |
| 12 | GB2485 | GB | Ⅳ | Female | 29 | Right temporal | PR | Yes | Yes |
| 13 | GB4860 | GB | Ⅳ | Female | 46 | Right temporal | GTR | Yes | Yes |
| 14 | GB0740 | GB | Ⅳ | Male | 49 | Right temporal | GTR | Yes | Yes |
| 15 | GB7686 | GB | Ⅳ | Male | 44 | Right parietal | GTR | Yes | Yes |
| 16 | GB5460 | GB | Ⅳ | Female | 53 | Right parietal | GTR | Yes | Yes |
| 17 | GB6447 | GB | Ⅳ | Male | 34 | Right paracele | GTR | Yes | Yes |
| 18 | GB6642 | GB | Ⅳ | Male | 29 | Right occipital | GTR | Yes | Yes |
| 19 | GB8407 | GB | Ⅳ | Male | 68 | Right frontal-temporal | GTR | Yes | Yes |
| 20 | GB2604 | GB | Ⅳ | Female | 36 | Right frontal-parietal | GTR | Yes | Yes |
| 21 | GB9016 | GB | Ⅳ | Male | 43 | Right frontal-parietal | GTR | Yes | Yes |
| 22 | GB8941 | GB | Ⅳ | Male | 69 | Right frontal-callosum | PR | Yes | Yes |
| 23 | GB3052 | GB | Ⅳ | Female | 38 | Right frontal | GTR | Yes | Yes |
| 24 | GB8664 | GB | Ⅳ | Female | 72 | Right frontal | GTR | Yes | Yes |
| 25 | GB0930 | GB | Ⅳ | Male | 67 | Right frontal | PR | Yes | Yes |
| 26 | GB3802 | GB | Ⅳ | Female | 62 | Right frontal | GTR | Yes | Yes |
| 27 | GB9428 | GB | Ⅳ | Female | 50 | Right frontal | GTR | Yes | Yes |
| 28 | GB1587 | GB | Ⅳ | Female | 39 | Right frontal | GTR | Yes | Yes |
| 29 | GB9816 | GB | Ⅳ | Female | 59 | Right frontal | GTR | Yes | Yes |
| 30 | GB3786 | GB | Ⅳ | Male | 44 | Right frontal | GTR | Yes | Yes |
| 31 | GB8396 | GB | Ⅳ | Male | 63 | Left temporal-parietal | PR | Yes | Yes |
| 32 | GB6547 | GB | Ⅳ | Female | 45 | Left temporal-parietal | GTR | Yes | Yes |
| 33 | GB6987 | GB | Ⅳ | Male | 68 | Left temporal | GTR | Yes | Yes |
| 34 | GB2640 | GB | Ⅳ | Male | 43 | Left parietal | GTR | Yes | Yes |
| 35 | GB1308 | GB | Ⅳ | Female | 44 | Left parietal | GTR | Yes | Yes |
| 36 | GB0052 | GB | Ⅳ | Female | 60 | Left frontal-temporal | PR | Yes | Yes |
| 37 | GB1401 | GB | Ⅳ | Male | 36 | Left frontal-temporal | PR | Yes | Yes |
| 38 | GB2199 | GB | Ⅳ | Male | 41 | Left frontal-temporal | GTR | Yes | Yes |
| 39 | GB7466 | GB | Ⅳ | Male | 41 | Left frontal | GTR | Yes | Yes |
| 40 | GB3249 | GB | Ⅳ | Male | 45 | Left frontal | PR | Yes | Yes |
| 41 | GB9842 | GB | Ⅳ | Male | 44 | Left frontal | GTR | Yes | Yes |
| 42 | GB0898 | GB | Ⅳ | Male | 51 | Left frontal | GTR | Yes | Yes |

Abbreviations: GB, Glioblastoma; GTR, Gross total resection; PR, Partial resection.

**Supplementary Table 2** Primers used for qRT-PCR.

| Gene name | Forward Primer Sequence (5' - 3') | Reverse Primer Sequence (5' - 3') |
| --- | --- | --- |
| *ATG5* | AAAGATGTGCTTCGAGATGTGT | CACTTTGTCAGTTACCAACGTCA |
| *ATG7* | CAGTTTGCCCCTTTTAGTAGTGC | CCAGCCGATACTCGTTCAGC |
| *LC3B* | GAGAAGCAGCTTCCTGTTCTGG | GTGTCCGTTCACCAACAGGAAG |
| *BECN1* | GGTGTCTCTCGCAGATTCATC | TCAGTCTTCGGCTGAGGTTCT |
| *HMGB1* | TATGGCAAAAGCGGACAAGG | CTTCGCAACATCACCAATGGA |
| *CD68* | GGAAATGCCACGGTTCATCCA | TGGGGTTCAGTACAGAGATGC |
| *IFNA* | GTCCAACGCAAAGCAATACA | ATATTGCAGGCAGGACAACC |
| *INOS* | GCTCTACACCTCCAATGTGACC | CTGCCGAGATTTGAGCCTCATG |
| *TNFA* | CCTGTGAGGAGGACGAACAT | GGTTGAGGGTGTCTGAAGGA |
| *ARG1* | TGGACAGACTAGGAATTGGCA | CCAGTCCGTCAACATCAAAACT |
| *CD163* | GCGGGAGAGTGGAAGTGAAAG | GTTACAAATCACAGAGACCGCT |
| *CD206* | AGCCAACACCAGCTCCTCAAGA | CAAAACGCTCGCGCATTGTCCA |
| *CSF2* | TCCTGAACCTGAGTAGAGACAC | TGCTGCTTGTAGTGGCTGG |
| *CCL2* | AGAATCACCAGCAGCAAGTGTCC | TCCTGAACCCACTTCTGCTTGG |
| *IL-1B* | ATGATGGCTTATTACAGTGGCAA | GTCGGAGATTCGTAGCTGGA |
| *IL-6* | ACTCACCTCTTCAGAACGAATTG | CCATCTTTGGAAGGTTCAGGTTG |
| *CCL22* | ATCGCCTACAGACTGCACTC | GACGGTAACGGACGTAATCAC |
| *VEGF* | AGGGCAGAATCATCACGAAGT | AGGGTCTCGATTGGATGGCA |
| *Ccl2* | TTAAAAACCTGGATCGGAACCAA | GCATTAGCTTCAGATTTACGGGT |
| *Il-1b* | GCAACTGTTCCTGAACTCAACT | ATCTTTTGGGGTCCGTCAACT |
| *Il-6* | TAGTCCTTCCTACCCCAATTTCC | TTGGTCCTTAGCCACTCCTTC |
| *Ptgs2* | TGAGCAACTATTCCAAACCAGC | GCACGTAGTCTTCGATCACTATC |
| *Tnfa* | CCCTCACACTCAGATCATCTTCT | GCTACGACGTGGGCTACAG |
| *Arg1* | TTGGGTGGATGCTCACACTG | GTACACGATGTCTTTGGCAGA |
| *Ccl24* | ATTCTGTGACCATCCCCTCAT | TGTATGTGCCTCTGAACCCAC |
| *Ccr2* | ATCCACGGCATACTATCAACATC | CAAGGCTCACCATCATCGTAG |
| *Cd200r1* | AGGCATTTCCAGTATCACAAGG | CCAATGGCCGACAAAGTAAGG |
| *Chil3* | GTACAAGCTGGTCTGCTACTTC | ATGTGCTAAGCATGTTGTCGC |
| *Mrc1* | CTCTGTTCAGCTATTGGACGC | CGGAATTTCTGGGATTCAGCTTC |
| *Pparg* | GGAAGACCACTCGCATTCCTT | GTAATCAGCAACCATTGGGTCA |
| *TLR2* | ATCCTCCAATCAGGCTTCTCT | GGACAGGTCAAGGCTTTTTACA |
| *TLR4* | AGACCTGTCCCTGAACCCTAT | CGATGGACTTCTAAACCAGCCA |
| *TLR9* | CTGCCTTCCTACCCTGTGAG | GGATGCGGTTGGAGGACAA |
| *RAGE* | GTGTCCTTCCCAACGGCTC | ATTGCCTGGCACCGGAAAA |
| *Tlr2* | CTCTTCAGCAAACGCTGTTCT | GGCGTCTCCCTCTATTGTATTG |
| *Tlr4* | GCCTTTCAGGGAATTAAGCTCC | GATCAACCGATGGACGTGTAAA |
| *Tlr9* | ATGGTTCTCCGTCGAAGGACT | CAGGTGGTGGATACGGTTGG |
| *Rage* | GCCACTGGAATTGTCGATGAGG | GCTGTGAGTTCAGAGGCAGGAT |
| *Hmgb1* | GCTGACAAGGCTCGTTATGAA | CCTTTGATTTTGGGGCGGTA |
| *GAPDH* | CTGCACCACCAACTGCTTAG | AGGTCCACCACTGACACGTT |
| *Actb* | GGCTGTATTCCCCTCCATCG | CCAGTTGGTAACAATGCCATGT |

**Supplementary Table 3** The sequences of shRNA.

| Vector | Sequence (5' to 3') |
| --- | --- |
| sh*Ctrl* | TTCTCCGAACGTGTCACGTAA |
| sh*HMGB1*-1 | CCCAGATGCTTCAGTCAACTT |
| sh*HMGB1*-2 | CCGTTATGAAAGAGAAATGAA |
| sh*HMGB1*-3 | GAAGAAGATGAAGATGAAGAA |

| Vector | Sequence (5' to 3') |
| --- | --- |
| sh*Ctrl* | TTCTCCGAACGTGTCACGTAA |
| sh*Hmgb1*-1 | CGGCCTTCTTCTTGTTCTGTT |
| sh*Hmgb1*-2 | TGACAAGGCTCGTTATGAAAG |
