# Supplementary material for: Autophagy-based unconventional secretion of HMGB1 in glioblastoma promotes chemosensitivity to temozolomide through macrophage M1-like polarization
Source: J Exp Clin Cancer Res. 2022 Feb 22;41:74. doi: 10.1186/s13046-022-02291-8 (PMC8862393; doi:10.1186/s13046-022-02291-8)
Supplement: Supplementary file 10 — Additional file 10: Figure S10. The expression of cytokines released by the activation of RAGE-NFκB-NLRP3 inflammasome pathway in extracellular NLRP3-enriched regions of GL261 cell-derived xenograft tumors. [file 13046_2022_2291_MOESM10_ESM.pdf]

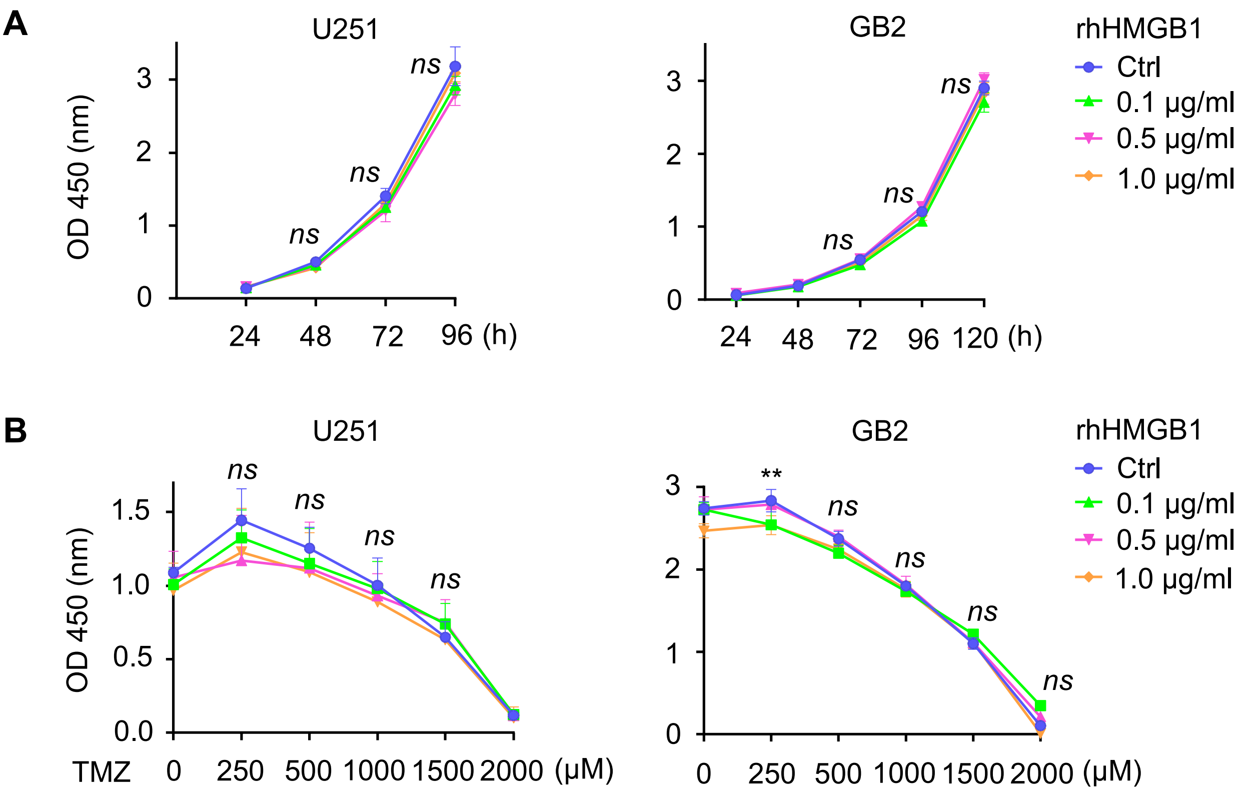
A
U251
GB2
OD 450 (nm)
ns
24
48
72
96
120
(h)
rhHMGB1
Ctrl
0.1 μg/ml
0.5 μg/ml
1.0 μg/ml
B
**
TMZ
0
250
500
1000
1500
2000
(μM)

Figure S10
